# Tripartite motif 31 drives gastric cancer cell proliferation and invasion through activating the Wnt/β-catenin pathway by regulating Axin1 protein stability

**DOI:** 10.1038/s41598-023-47139-z

**Published:** 2023-11-16

**Authors:** Qi Feng, Fengting Nie, Lihong Gan, Xianpin Wei, Peng Liu, Hui Liu, Kaige Zhang, Ziling Fang, Heng Wang, Nian Fang

**Affiliations:** 1 Department of Gastroenterology, The Third Affiliated Hospital of Nanchang University Or Nanchang First Hospital, 128 Xiangshan North Road, Nanchang, 330008 Jiangxi People’s Republic of China; 2 Department of Oncology, The First Affiliated Hospital of Nanchang University, 1519 Dongyue Avenue, Nanchang, 330006 Jiangxi People’s Republic of China; 3 Department of Orthopedics, The First Affiliated Hospital of Nanchang University, 1519 Dongyue Avenue, Nanchang, 330006 Jiangxi People’s Republic of China

**Keywords:** Cancer, Gastroenterology

## Abstract

Mounting evidence has proposed the importance of the Wnt/β-catenin pathway and tripartite motif 31 (TRIM31) in certain malignancies. Our research aimed to clarify the correlation between aberrant TRIM31 expression and the Wnt/β-catenin pathway during gastric cancer (GC) oncogenesis and development. TRIM31 was drastically elevated in GC tissues and was closely associated with aggressive clinical outcomes and poor prognosis. Moreover, TRIM31 downregulation attenuated GC cell proliferation and invasion in vitro. Mechanistically, TRIM31 could bind and ubiquitinate Axin1 protein, thereby facilitating the activation of the Wnt/β-catenin pathway. Additionally, Axin1 knockdown partially abrogated the inhibitory effects on the proliferative, invasive and migratory abilities of GC cells induced by TRIM31 silencing. Furthermore, TRIM31 was negatively correlated with Axin1 protein expression in GC tissues. In summary, we revealed a new TRIM31-Axin1-Wnt/β-catenin axis that contributed greatly to the progression of GC, and targeting this regulatory axis may represent an effective treatment for GC patients.

## Introduction

Gastric cancer (GC) is a major malignant cancer and contributes to a heavy social burden globally owing to its increasing prevalence^1,2^. The overall survival of patients with GC is poor due to its high recurrence and metastasis rates^3,4^. Hence, it is of importance to elucidate the mechanisms and pathological processes underlying GC tumorigenesis and development.

The Wnt/β-catenin signaling pathway has been shown to play a fundamental role in multiple biological and pathological processes, including human cancers^5^. When the Wnt/β-catenin pathway is stimulated, intracellular β-catenin rapidly accumulates, and translocates into the nucleus to facilitate cyclin D1 and c-Myc transcription, resulting in cell proliferation and metastasis^6,7^. While the Wnt signaling pathway is absent, the accumulated β-catenin is phosphorylated by the destructive complex (APC-Axin1/2-GSK-3β)^7^. Next, the cytoplasmic β-catenin will be ubiquitinated for the degradation through the ubiquitin–proteasome system. As its dysregulation contributes greatly to several cancers, including GC, further studies are needed to investigate the complicated mechanisms underlying the activation of Wnt/β-catenin pathway.

Tripartite motif 31 (TRIM31), a member of the TRIM protein family, is an E3 ubiquitin ligase and serves as an important regulator of carcinogenesis and the progression of several cancers owing to its role in promoting substrate degradation and activating signal transduction through ubiquitin modifications^8–10^. Some studies have demonstrated that TRIM31 is involved in tumor growth and metastasis in nasopharyngeal carcinoma, glioblastoma, pancreatic and colorectal cancer^11–14^. TRIM31 promotes liver cancer progression by degrading the tuberous sclerosis complex (TSC) 1 and TSC2 complex and activating the mammalian target of rapamycin complex1 signaling pathway^15^. Other dysregulated signaling pathways are responsible for TRIM31 functions including NF-κB and Akt pathways, which are tightly associated with cancer cell proliferation and invasion^9,14,16^. More interestingly, TRIM31 overexpression has been reported in early GC tissues^17,18^. Additionally, TRIM31 is also as a positive activator of the Wnt/β-catenin pathway in acute myeloid leukemia^19^. However, the exact correlation between the Wnt/β-catenin pathway and TRIM31 in GC remains unknown.

The aim of our study is to unveil the correlation between TRIM31 and Wnt/β-catenin signaling in GC tumorigenesis and progression. Our data show that TRIM31 overexpression activates the Wnt/β-catenin pathway by promoting the ubiquitination and degradation of Axin1 protein, and thereby providing novel insights into the treatments for GC patients.

## Results

### Upregulated TRIM31 expression in GC

**Figure 1:**
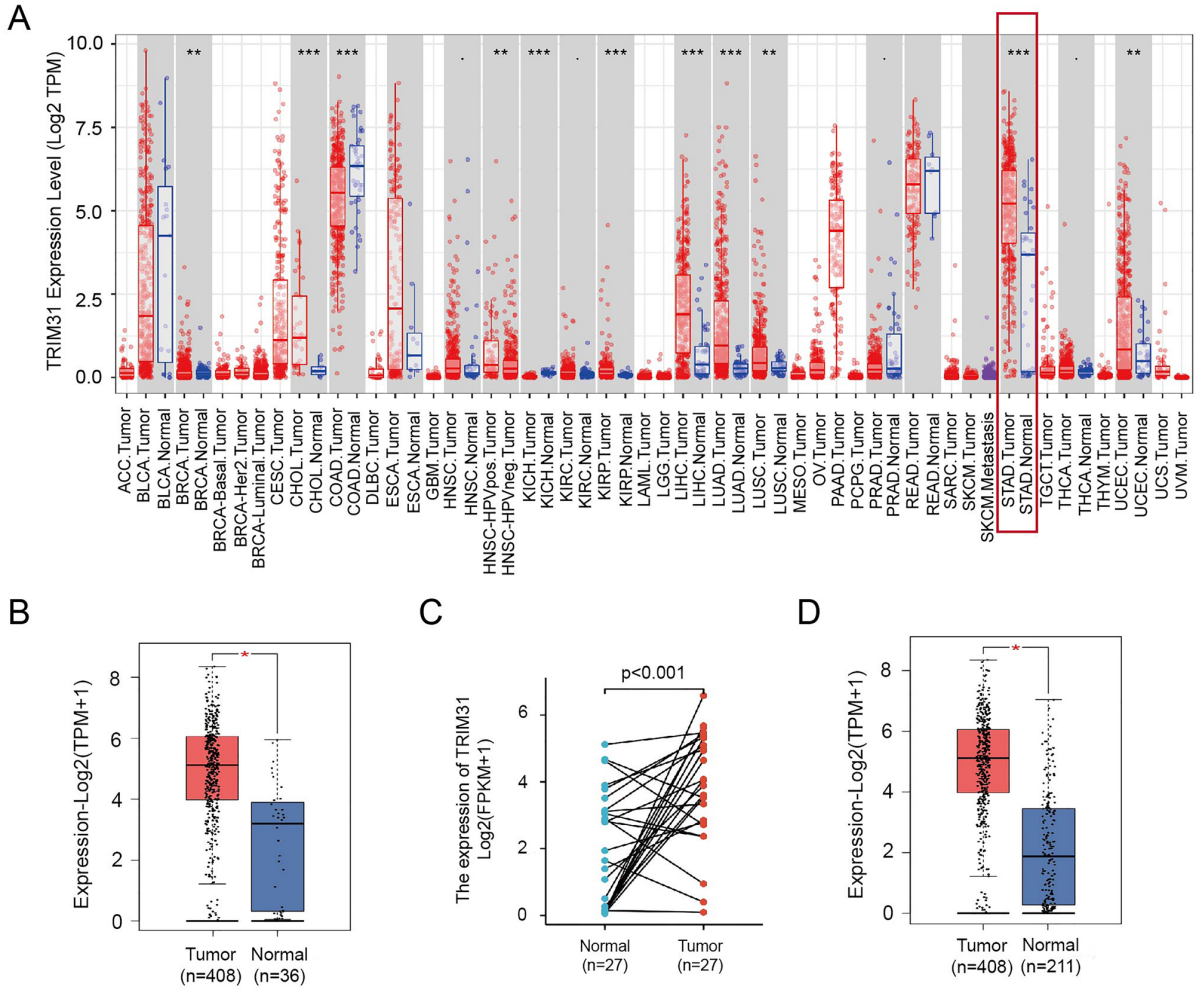
Upregulated TRIM31 expression in Gastric Cancer. (**A**) Human TRIM31 expression levels were determined in 38 types of cancers using TIMER database (including GC, represented in the red frame). (**B**) Bar chart of the quantified relative expression profile of TRIM31 in GC tissues (displayed in red, n = 408) in comparison to non-cancerous normal tissues (marked in blue, n = 36) in TCGA database (*P < 0.05). (**C**) Higher TRIM31 expression levels in GC tissues (n = 27) are indicated in red, while lower levels in their paired para-cancerous normal tissues of TCGA database are indicated in blue (controls; n = 27, P < 0.001). (**D**) Bar graph of TRIM31 expression in GC tissues (red, n = 408) from TCGA database and in non-cancerous normal tissues (blue, n = 211) from TCGA and GTEx databases (*P < 0.05).

To determine TRIM31 expression in various cancers, preliminary screening was conducted via TIMER^20^ (Tumor Immune Estimation Resource, http://timer.cistrome.org/) and GEPIA2^21^ (Gene Expression Profiling Interactive Analysis, http://gepia2.cancer-pku.cn/) online tools. As illustrated in Fig. 1A, TRIM31 was significantly overexpressed in several tumor types, including GC. To determine the exact expression of TRIM31 in GC, we used online databases for subsequent analysis. Using the TCGA (the Cancer Genome Atlas) data portal, we observed that TRIM31 was overexpressed in GC tissues compared with non-cancerous tissues by GEPIA2 (Fig. 1B, P < 0.05), and TRIM31 expression levels were upregulated in GC tissues compared to the paired normal tissues by R software (Fig. 1C, P < 0.001). Data from TCGA and Genotype-Tissue Expression (GTEx) also demonstrated that TRIM31 expression was drastically increased in GC tissues compared with the non-cancerous tissues by GEPIA2 (Fig. 1D, P < 0.05). Taken together, these data suggested that TRIM31 was overexpressed in GC.

### Higher TRIM31 expression predicts disease progression and worse prognosis

**Figure 2:**
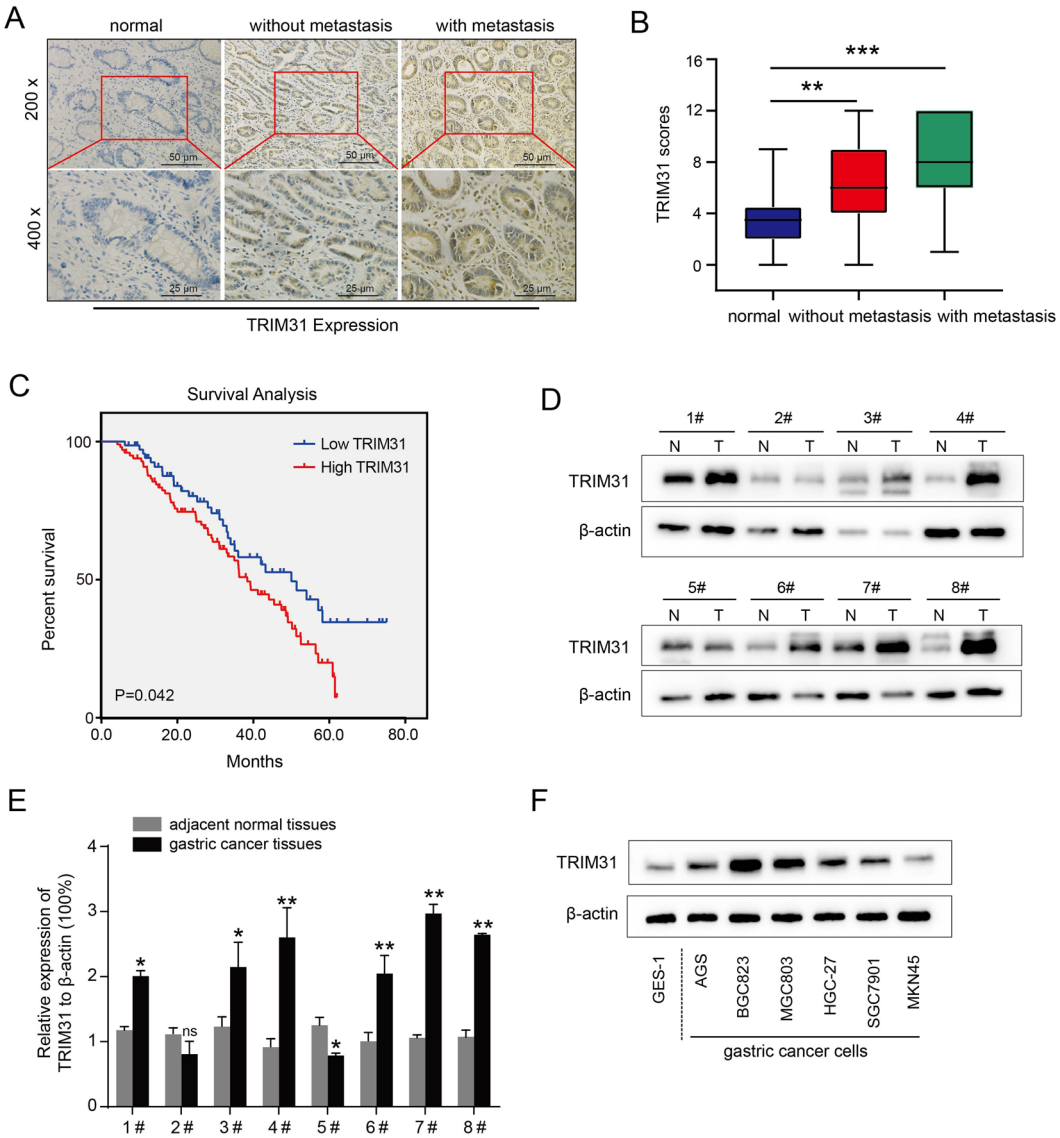
Higher TRIM31 expression predicts disease progression and worse prognosis. (**A**) Representative IHC staining photographs of TRIM31 expression were obtained from GC tissues (n = 170) and their corresponding non-cancerous tissues (n = 46). (**B**) Relative quantification of TRIM31 immunostaining scores. (**C**) Kaplan–Meier survival curve of all enrolled GC patients (P < 0.05). (**D**,**E**) Western blotting assays of TRIM31 expression in 8 GC tissues and their corresponding para-cancerous normal tissues. Bar graph of quantified relative TRIM31 expression normalized to β-actin expression. (**F**) TRIM31 expression in GES-1 and six GC cell lines determined by western blotting (*P < 0.05 and **P < 0.01).

As TRIM31 was predicted to correlate with GC tumorigenesis and progression, we further examined the expression levels and clinical significance of TRIM31 in GC specimens. In total, 170 paraffin-embedded GC tissue samples were collected for IHC staining. As shown in Fig. 2A, TRIM31 was frequently overexpressed in samples of GC patients (99/170, 58.2%). TRIM31 upregulation was positively associated with the TNM stage (P = 0.021), depth of invasion (P = 0.003), and lymph node metastasis (P = 0.041) (Table 1). Further analysis revealed that the staining scores of TRIM31 in the metastasis group were markedly higher than those in the non-metastasis group (Fig. 2A,B, P < 0.05). Additionally, the prognosis of patients exhibiting high TRIM31 expression was much inferior than that of patients exhibiting low expression, indicating that TRIM31 may serve as a prognostic indicator for GC patients (Fig. 2C). The AUC (area under the ROC curve) value of ROC (receiver operating characteristic) analysis of TRIM31 overexpression was 0.75, and the AUC value of time-dependent ROC of TRIM31 at 1, 3, 5 years was 0.91, 0.79 and 0.65, respectively, which could also enhance the significance of TRIM31 overexpression as a prognosis marker for GC patients during the 5-year follow-up (Fig. S1). Similarly, TRIM31 was highly expressed in fresh gastric cancerous tissues compared to their corresponding normal tissues (6/8, 75%, Fig. 2D,E). Furthermore, TRIM31 expression was upregulated in GC cell lines, such as BGC823 and HGC-27, compared to normal gastric epithelial cells (GES-1), as determined by western blotting analysis (Fig. 2F and Fig. S2). In summary, TRIM31 was overexpressed in GC tissues and cell lines as a predictive factor for GC progression and prognosis.

**Table 1:** The connection between TRIM31 expression levels and clinical parameters of GC patients.

Clinicopathological parameters	n = 170	TRIM31 expression (%)		P value
		Low n = 71 (41.8)	High n = 99 (58.2)	
Age (years)				
< 65	101	47 (46.5)	54 (53.5)	0.127
≥ 65	69	24 (34.8)	45 (65.2)	
Sex				
Male	95	43 (45.3)	52 (54.7)	0.298
Female	75	28 (37.3)	47 (62.7)	
Tumor size (cm)				
< 5	94	44 (46.8)	50 (53.2)	0.138
≥ 5	76	27 (35.5)	49 (64.5)	
Differentiation				
Moderately or well	84	37 (44.0)	47 (56.0)	0.551
Poorly	86	34 (39.5)	52 (60.5)	
Depth of invasion				
T1–T2	78	42 (53.8)	36 (46.2)	**0.003**
T3–T4	92	29 (31.5)	63 (68.5)	
Lymph node metastasis				
With	73	37 (50.7)	36 (49.3)	**0.041**
Without	97	34 (35.1)	63 (64.9)	
TNM stage				
I–II	71	37 (52.1)	34 (47.9)	**0.021**
III–IV	99	34 (34.3)	65 (65.7)	

The chi-square test was utilized for statistical analysis.

Significant values are in bold.

### TRIM31 depletion attenuates the proliferative and invasive capacities of GC cells

**Figure 3:**
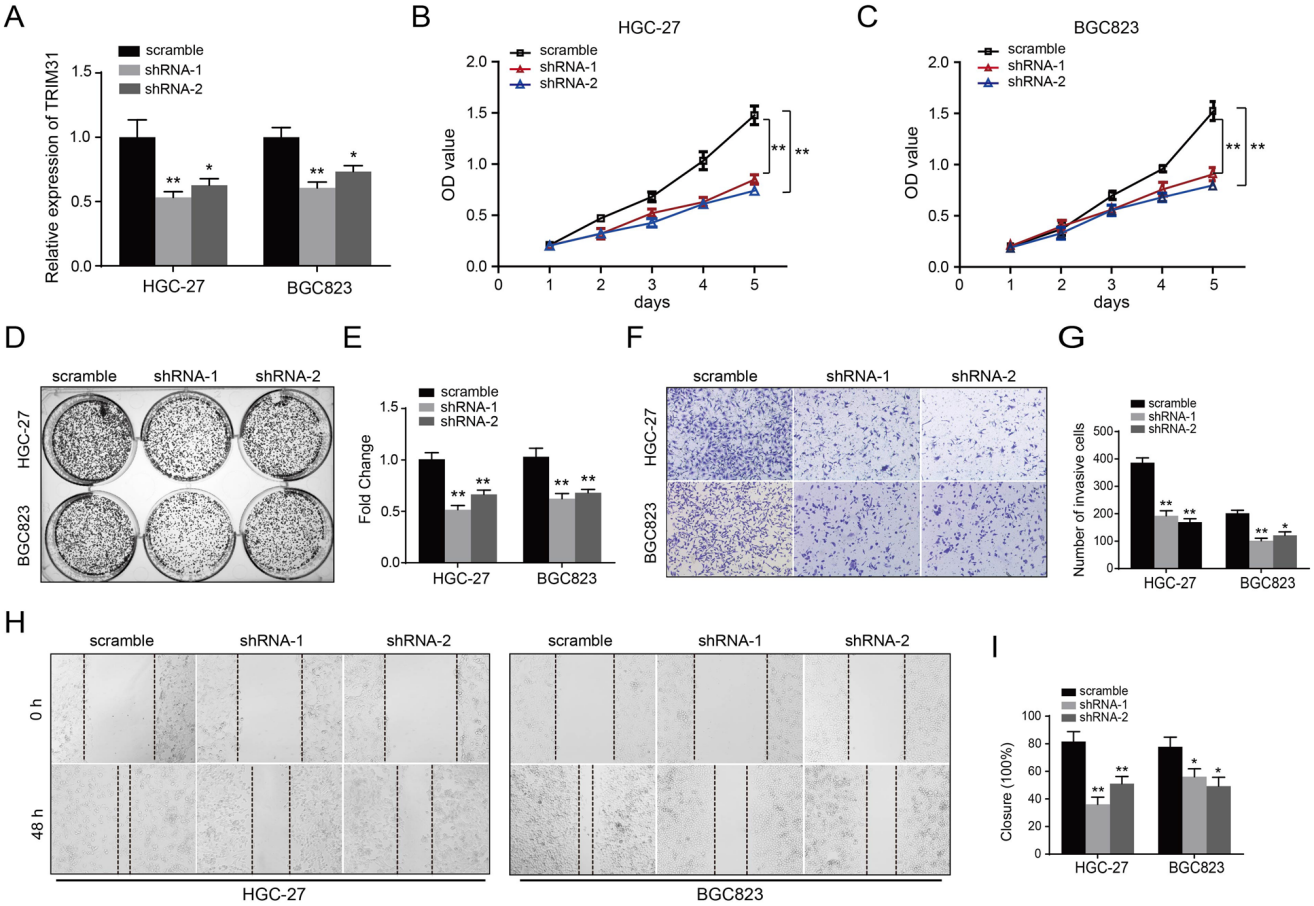
TRIM31 depletion attenuates the proliferative and invasive capacities of GC cells. (**A**) The mRNA expression of TRIM31 decreased upon TRIM31 knockdown in GC cells as determined by qRT-PCR assays. (**B**,**C**) OD values were detected by CCK-8 assays upon TRIM31 knockdown in HGC-27 and BGC823 cells. (**D**,**E**) Representative images of HGC-27 and BGC823 cells from colony-formation assays. Colony data are pictured in the bar graph. (**F**,**G**) Representative photographs and quantification of GC cells as indicated in transwell experiments (magnification, × 200). (**H**,**I**) The migration rate of GC cells upon TRIM31 knockdown detected by wound-healing assays (one‐way ANOVA or t-test, *P < 0.05 and **P < 0.01, respectively).

We conducted qRT-PCR, CCK-8, colony-forming, transwell and wound healing assays to assess the biological functions of TRIM31 in GC cells. qRT-PCR assays were performed to confirm the efficiency of shRNAs targeting TRIM31 (Fig. 3A). The growth curves showed that TRIM31 knockdown greatly slowed down HGC-27 and BGC823 cell proliferation by CCK-8 assays (Fig. 3B,C). These data were further validated by colony formation experiments (Fig. 3D,E). Additionally, transwell and wound healing assays revealed that TRIM31 knockdown inhibited the invasive and migratory abilities of GC cells (Fig. 3F–I). These data indicated that TRIM31 depletion attenuate the proliferation, invasion, and migration of GC cells.

### TRIM31 activates the Wnt/β-catenin pathway by enhancing Axin1 ubiquitination and degradation

**Figure 4:**
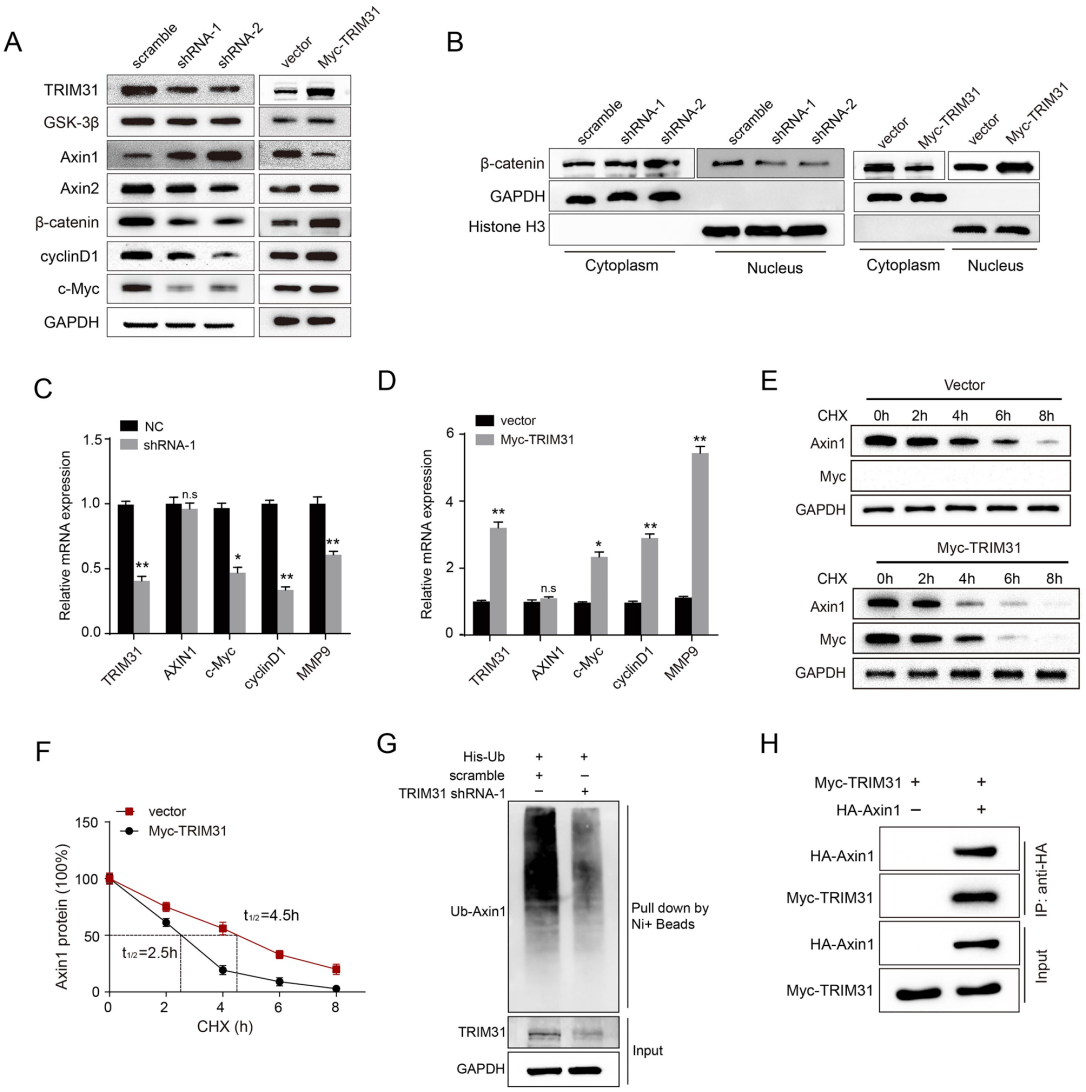
TRIM31 activates the Wnt/β-catenin pathway by enhancing Axin1 ubiquitination and degradation. (**A**) Relative protein expression levels of core Wnt/β-catenin pathway components and TRIM31 upon transfection in HGC-27 cells, as determined by western blotting assays. (**B**) The cytoplasmic/nuclear fractionation of β-catenin protein under TRIM31-knockdown and overexpression. GAPDH served as the cytoplasmic marker, while Histone H3 functioned as the nuclear marker. (**C**,**D**) The mRNA expression of TRIM31 and core components of the Wnt/β-catenin signaling pathway detected via qRT-PCR assays using modified HGC-27 cells after transfection. (**E**) Axin1 protein expression in modified HGC-27 cells with 100 μg/ml CHX at the indicated time points. (**F**) Line graph of Axin1 protein expression standardized with GAPDH control group at corresponding time points. (**G**) The in vivo ubiquitination assays were performed to detect the Axin1 protein expression upon TRIM31 knockdown. Indicated antibodies were utilized for detecting the input and binding proteins using WB analysis. (**H**) Interaction between TRIM31 and Axin1 in HGC-27 cells via co-IP assays (*ns* no significance, *P < 0.05 and **P < 0.01).

Our previous research demonstrated that the Wnt/β-catenin signaling pathway plays a crucial role in GC tumorigenesis and development^22,23^. Therefore, we further explored whether the Wnt/β-catenin pathway was accountable for TRIM31 function in GC. TRIM31 deficiency decreased the protein expression of several essential elements of the Wnt/β-catenin pathway, such as c-Myc, cyclinD1 and β-catenin, without any significant effects upon Axin2 or GSK-3β expression (Fig. 4A and Fig. S3). In addition, the ectopic expression of TRIM31 exerted opposite effects. More convincingly, the cytoplasmic/nuclear fractionation assays showed that overexpression of TRIM31 significantly increases the expression of nuclear β-catenin protein. Conversely, TRIM31 knockdown revealed opposite effects (Fig. 4B). Notably, we found that Axin1 protein expression was markedly changed upon TRIM31 depletion or overexpression. Based on the qRT-PCR data (Fig. 4C,D), the mRNA levels of c-Myc, cyclinD1 and matrix metalloproteinase 9 (MMP9) were correlated with upregulation and downregulation of TRIM31 in GC cells, without affecting Axin1 mRNA expression. These findings indicated that TRIM31 regulates Axin1 protein expression at the post-transcriptional level. Therefore, we next attempted to use CHX-chase assay to clarify the mechanism involved in the TRIM31-mediated regulation of Axin1. As expected, the chase experiments showed that the half-time decay of Axin1 protein dramatically declined in TRIM31-overexpressed cells (Fig. 4E,F, 4.5 h vs. 2.5 h, P < 0.05). Furthermore, we found that TRIM31 could bind to Axin1 specifically, thus leading to the ubiquitination and degradation of Axin1 protein in GC cells (Fig. 4G,H). The aforementioned data suggested that TRIM31 bind and destabilize the Axin1 protein, thereby orchestrating the Wnt/β-catenin pathway in GC cells.

### Axin1 silencing rescues the proliferation and invasion of TRIM31 knockdown in GC cells

**Figure 5:**
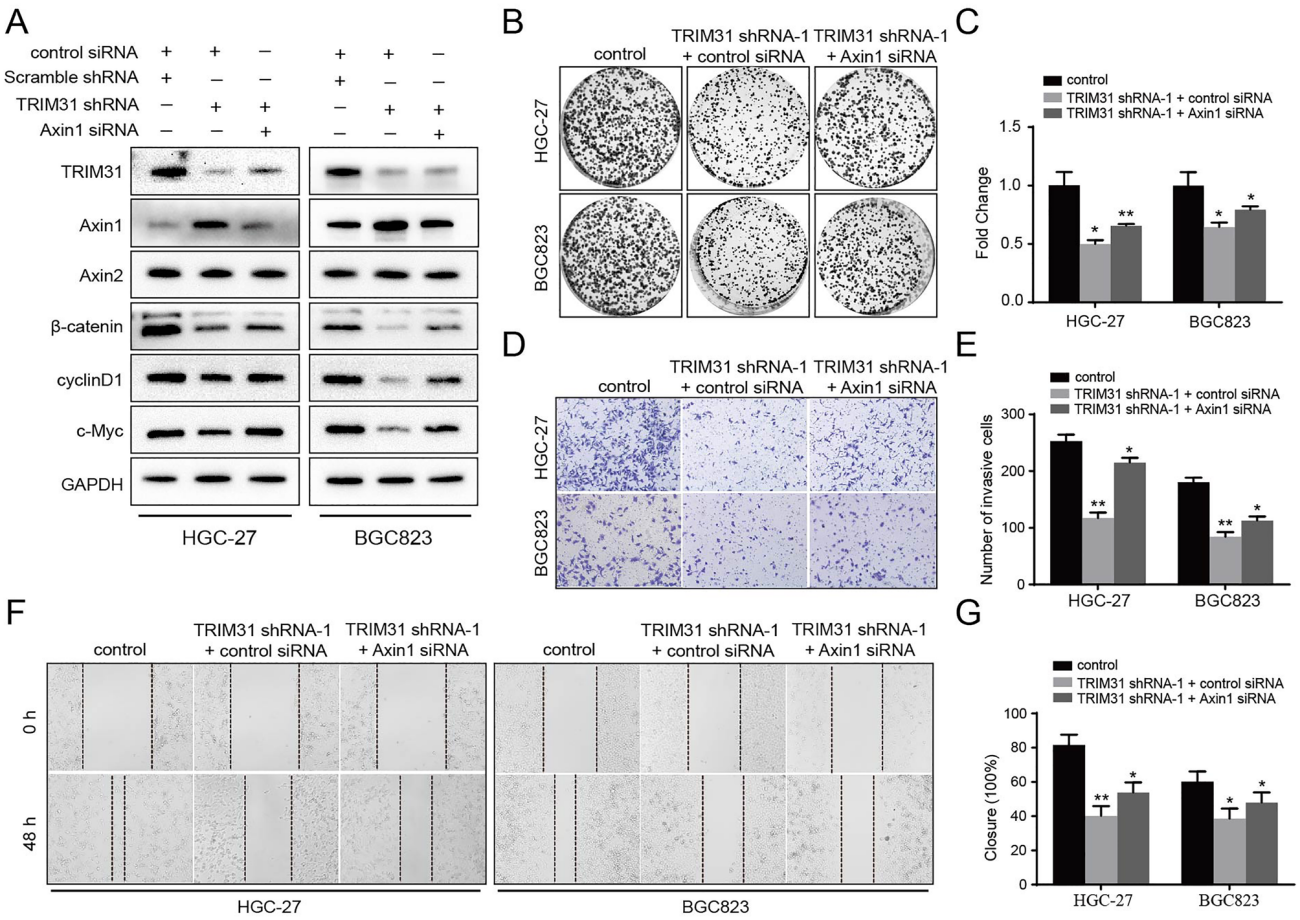
Axin1 silencing rescues the proliferation and invasion of TRIM31 knockdown in GC cells. (**A**) Relative protein expression levels of TRIM31, Axin1, and main Wnt pathway components were analyzed through WB assays upon transfection. (**B**,**C**) Representative images and relative quantification of HGC-27 and BGC823 cells from colony-formation experiments. (**D**,**E**) Representative images of GC cells from transwell invasion analysis (magnification, × 200). Bar graphs of the invasion data. (**F**,**G**) Wound healing assays revealed the migratory capabilities of modified GC cells. The relative quantification was shown in the bar graph (One-way ANOVA: *P < 0.05 and **P < 0.01).

To further confirm our hypothesis regarding Axin1 regulation by TRIM31 in GC cells, we evaluated Axin1 siRNA with or without TRIM31 shRNA in GC cells in colony formation, wound healing, and transwell assays. As shown in Fig. 5A, Axin1 knockdown partially negates the downregulation of β-catenin, c-Myc, and cyclinD1 induced by TRIM31 knockdown. Moreover, co-transfection of TRIM31 shRNA and Axin1 siRNA, the nuclear part of β-catenin protein increased markedly when compared with only TRIM31 shRNA group (Fig. S4). More strikingly, the TRIM31-mediated proliferative, migratory and invasive properties were partly abolished upon Axin1 deficiency, supported by colony formation, wound healing, and transwell assays (Fig. 5B–G, P < 0.05). Besides, we overexpressed TRIM31 with Axin1 siRNA or control siRNA, and then found a more significantly stimulative effect on Wnt/β-catenin pathway to promote proliferation, invasion and migration of GC cells (Fig. S5). Collectively, these assays elucidated that Axin1 is a fundamental contributor to TRIM31-induced oncogenesis in GC cells.

### TRIM31 was negatively correlated with Axin1 protein expression in GC tissues

**Figure 6:**
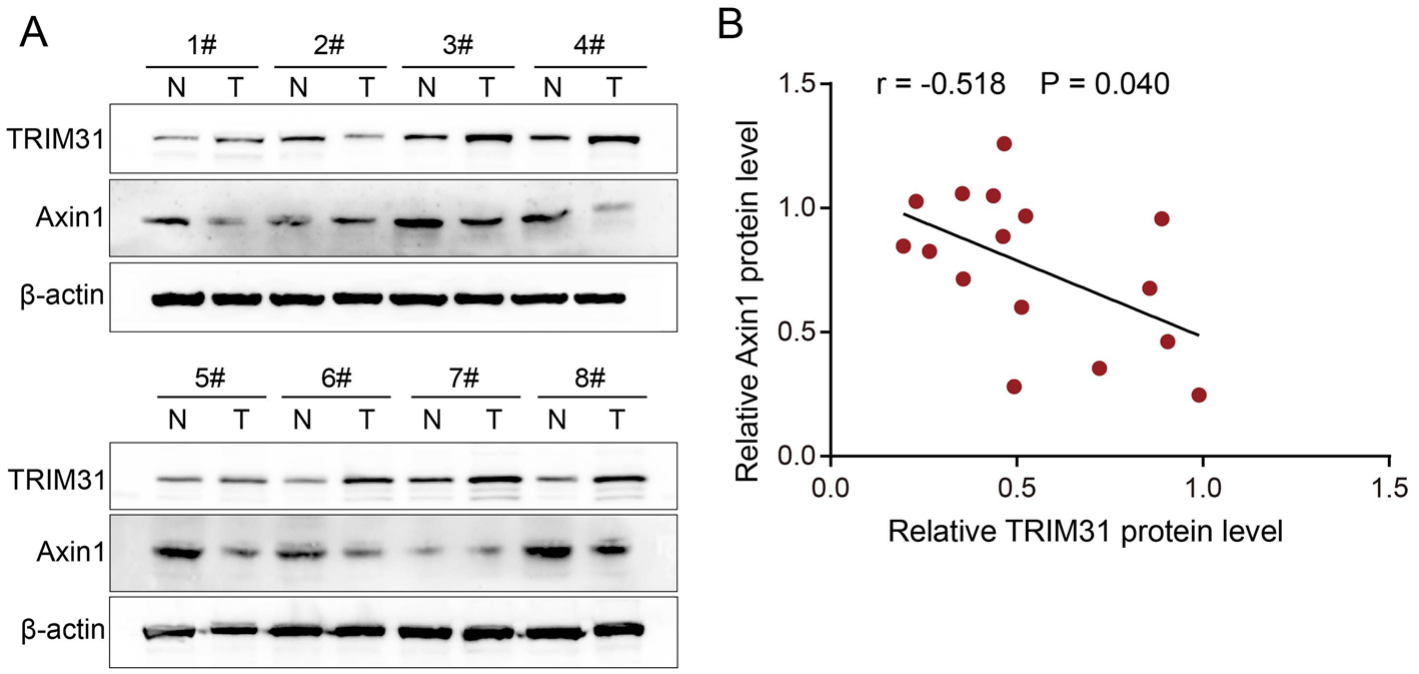
TRIM31 was negatively correlated with Axin1 protein expression in GC tissues. (**A**) Analysis of TRIM31 and Axin1 expression in 8 freshly collected GC tissues using western blotting assays. (**B**) Expression analysis of TRIM31 and Axin1 protein levels in 8 GC patients by the Pearson correlation test.

To further validate the relevance of Axin1 regulation of TRIM31 in GC, we performed the correlation analysis of TRIM31 and Axin1 protein expression in GC tissues. As shown in Fig. 6A, we found Axin1 expression were decreased in GC tissues when compared to non-cancerous tissues (5/8, 62.5%). Moreover, we also detected a significantly negative correlation between TRIM31 and Axin1 protein expression in GC tissues by the Pearson correlation test (Fig. 6B, r = − 0.518, P = 0.04). These data revealed that TRIM31 expression was negatively associated with Axin1 protein expression in GC tissues.

## Discussion

**Figure 7:**
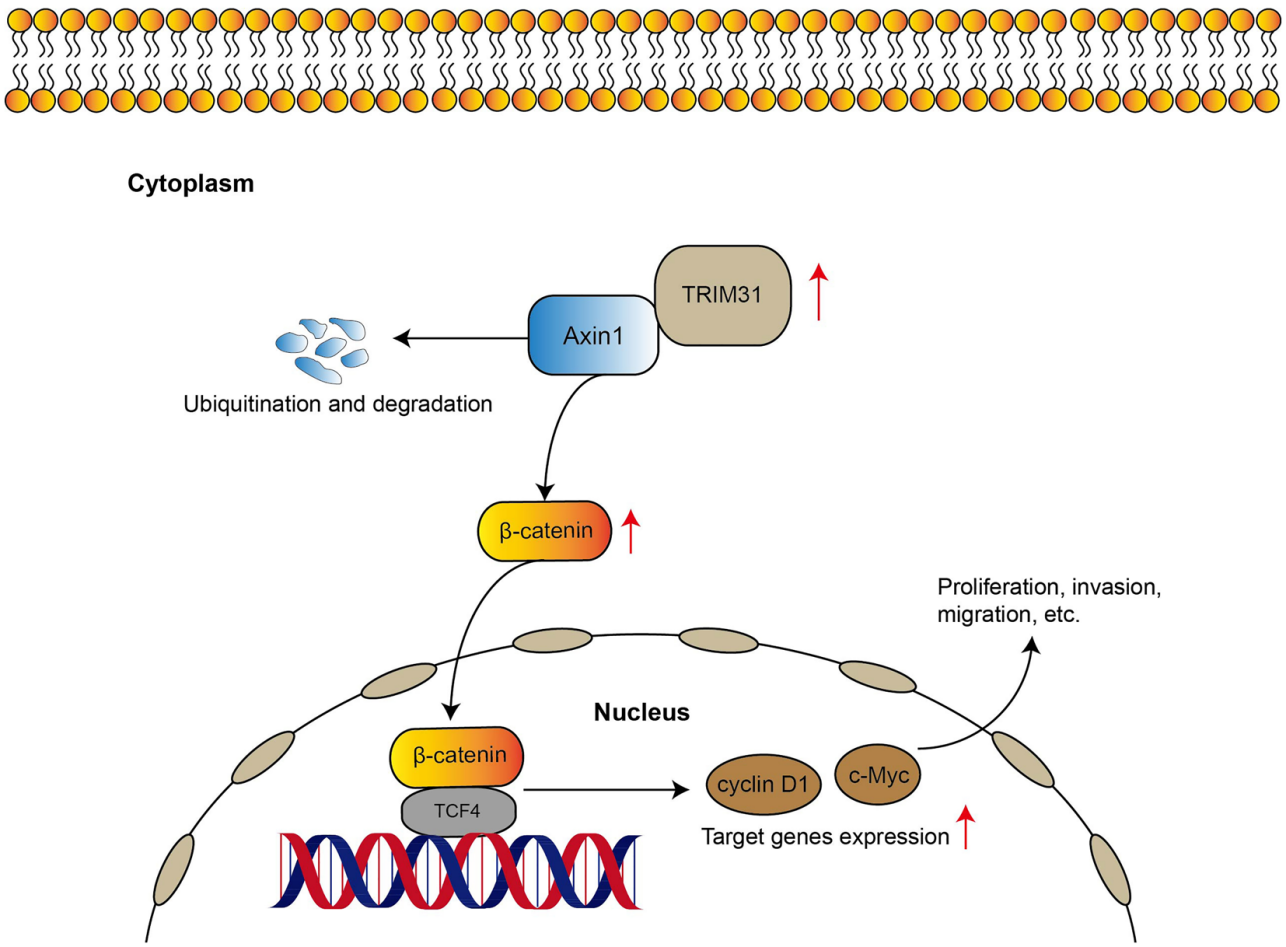
A model of TRIM31 regulation of Axin1-Wnt/β-catenin pathway in GC.

In this study, we found that TRIM31 facilitated cell proliferative and invasive abilities via orchestrating the Wnt/β-catenin pathway. TRIM31 was markedly upregulated in GC and its overexpression was related with aggressive phenotypes and prognosis in GC patients. Our mechanistic analysis revealed that TRIM31 interacted with and destabilized Axin1 protein stability through ubiquitination and degradation, thus activating the Wnt/β-catenin pathway, finally leading to GC tumorigenesis and progression. Hence, our study presented a tumor-promoting role of TRIM31 and a connection with Axin1 in GC, supporting a critical role of TRIM31-Axin1-β-catenin axis in GC development (Fig. 7).

Several studies have demonstrated that TRIM31 was elevated in several malignant carcinomas, such as nasopharyngeal neoplasms, glioblastomas, and colorectal cancers^11–13^. For instance, enhanced TRIM31 expression could be a positive biomarker for poor clinical outcomes, and TRIM31 confers gemcitabine resistance in pancreatic carcinoma cells by activating the NF-κB pathway^14^. However, TRIM31 is downregulated in non-small cell lung cancer and serves as a tumor suppressor^24^. These seemingly contradictory studies suggest that TRIM31 plays various roles in different cancer types. Results from other studies reveal that TRIM31 is overexpressed in GC tissues^17^; however, the biological effects and specific clinical significance of TRIM31 in GC are not determined. In this study, we firstly screened the expression profiles of TRIM31 using bioinformatics online database. And we found that TRIM31 was overexpressed in diverse tumors, including GC, further verified through a series of IHC and western blotting analysis. Moreover, our data revealed that increased TRIM31 expression was closely associated with disease progression and poor prognosis in GC patients. Furthermore, TRIM31 was responsible for the proliferation, migration, and invasion of GC cells, supported by a series of functional assays. These findings uncover that TRIM31 acts as a crucial oncogene in human GC, and the underlying mechanisms should be explored in depth in future research.

A bunch of literatures have shown that the Wnt/β-catenin signaling pathway is one of the most important pathways in GC tumorigenesis^25–30^. In this study, we defined TRIM31 as a cancer-promoting mediator that regulates the Wnt/β-catenin pathway. TRIM31 overexpression increased c-Myc, β-catenin, and cyclinD1 expression, while TRIM31 knockdown had the antagonized effect. Similarly, Xu et al. recently reported that TRIM31 boosted cancer progression and sensitivity to daunorubicin in acute myeloid leukemia by regulating Wnt/β-catenin signaling^19^; however, the exact regulatory mechanism involved remains unclear. Axin1, an essential element of the β-catenin destruction complex, negatively orchestrated the Wnt/β-catenin pathway in GC^31,32^. Our previous data have indicated that TRIM11 facilitates the activation of Wnt/β-catenin pathway by destabilizing Axin1^25^. Moreover, other TRIM family members, such as TRIM32, TRIM37 and TRIM65, could also function as positive regulators of Wnt/β-catenin pathway through similar mechanisms^33–37^. In this study, we found that TRIM31 bound and ubiquitinated Axin1 protein, thereby shortening the half-life of Axin1, consequently contributing to the stimulation of the Wnt/β-catenin pathway. More intriguingly, we found that TRIM31 may undergo ubiquitination itself, leading to proteasome-dependent degradation. Therefore, we suspect that the degradation of Axin1 might also happen at the level of the Axin1-TRIM31 complex by other unknown ubiquitin ligases, rather than merely depending on TRIM31, which should be further distinguished in our future study.

Furthermore, the influence of TRIM31 upon GC development would be partly negated through modulation of Axin1 expression, which further supporting that Axin1 functions as a predominant downstream factor of TRIM31. Axin1 depletion could not completely abolish TRIM31 depletion-induced effects in GC cells, suggesting that Axin1 was not a unique effector of TRIM31. Several studies have demonstrated that TRIM31 can manipulate cancer-related genes or pathways, such as the TSC1-TSC2 complex, p52 (Shc), MMP2/9, p53-AMPK axis, NF-κB and PI3K/Akt pathways^8,14,15,38–40^. Thus, it can be inferred that TRIM31 may also target these genes and pathways in GC. Altogether, our data not only present reliable evidence for regulating Axin1 expression in the Wnt/β-catenin pathway, but also indicate new therapeutic targets for treating GC patients.

## Conclusions

In summary, this study revealed a novel correlation between TRIM31 and the Wnt/β-catenin pathway. TRIM31 associated with and destabilized Axin1 protein to orchestrate the Wnt/β-catenin pathway, resulting in the tumorigenesis and progression of GC.

## Materials and methods

### Gene expression analysis

We use TIMER database to detect TRIM31 expression levels in multiple cancers. Then GEPIA2 database was utilized for TRIM31 expression in gastric cancerous and normal tissue from TCGA database or TCGA and GTE) databases. Next, we downloaded the gastric cancerous and corresponding non-cancerous tissues from TCGA online tool, which were dealt with R software version 4.2.1 for statistical analysis and visualization.

### Human specimens

We retrospectively collected 170 paraffin-embedded GC tissues from the Department of Pathology at the Third Affiliated Hospital of Nanchang University from 2017 to 2020. Demographics of patients from which the samples were obtained were as follows: 101 (59.4%) were aged < 65 years and 69 (40.6%) were aged ≥ 65 years, and 75 (44.1%) and 95 (55.9%) were female and male, respectively. None of the enrolled patients received treatment before surgery. The clinicopathological features of these 170 patients were sorted and analyzed by two pathologists (Table 1). Fresh GC samples and their corresponding normal tissues (8 pairs in total) were obtained from surgical operating room and immediately stored in liquid nitrogen.

### Cell lines and cell culture

Normal gastric epithelial cells and six human GC cell lines (AGS, MGC-803, SGC-7901, BGC-823, MKN-45, and HGC-27) were from the American Type Culture Collection (Manassas) and the Shanghai Institute of Cell Biology (China). All cell lines were raised in RPMI-1640 (Gibco, Carlsbad, CA, USA) or Dulbecco’s Modified Eagle Medium (DMEM) supplied with 10–15% fetal bovine serum (FBS, Gibco, USA) in 37 °C at 5% CO_2_.

### Western blotting analysis

Total proteins of GC cells and tissues were extracted using radioimmunoprecipitation assay (RIPA) lysis buffer (Sangon Biotech, China) with 1% protease inhibitor cocktail (CoWin Biosciences, China). Nuclear protein was extracted using Nuclei Isolation Kit (Sangon Biotech, China) according to the manufacturer’s instructions. All the proteins were separated using gradient SDS-PAGE and transferred to PVDF membranes. Membranes were blocked with TBST solution containing 5% skim milk powder at 25 °C. Due to different experimental purposes, the blots were cut prior to hybridization with antibodies. After incubation with primary antibodies at 4 °C overnight, the membranes were washed with TBST for 5 min each time, followed by incubation with secondary antibodies for 60 min. Finally, the protein bands were visualized using the chemiluminescence kit (Boster, China) and be analyzed through mage J software.

All primary antibodies used in this study were as follows: TRIM31 (1:2000, Abcam, ab67785), β-catenin (1:2000, BD, 610,153), Histone H3 (1:1000, CST, 4499), c-Myc (1:2000, Affinity, AF0358), GSK-3β (1:1000, CST, 9315), Axin1 (1:1000, Fitzgerald, 70R-51344), Axin2 (1:2000, Prosci, 6163), cyclinD1 (1:5000, Proteintech, 60186-1-Ig), β-actin (1:5000, Proteintech, 20536-1-AP) and GAPDH (1:5000, Proteintech, 60004-1-Ig). β-actin and GAPDH were used as negative controls.

### Cell counting Kit-8 (CCK-8) assay

Briefly, 5000 GC cells were seeded into a 96-well plate before transfection, as previously reported^41^. To each well, 10 μL CCK-8 reagent was added at 0, 24, 48, 72, or 96 h, and the plate was cultured at 37 °C for 1 h. The absorbance of these cells was evaluated at 450 nm according to the manufacturer’s instructions.

### Immunohistochemistry (IHC) analysis

The clinical specimens were stained using IHC assays as described previously^23^. The immunohistochemical evaluation of TRIM31 expression was conducted independently by two experienced pathologists. We multiplied the staining levels and positive ranges to calculate the staining values of the score index (SI) according to the previously described criteria^25^. High TRIM31 expression was defined as SI ≥ 6, and low TRIM31 expression levels were the rest of specimens.

### Quantitative real-time PCR (qRT-PCR) analysis

Total RNA was first collected from cells or tissues using TRIzol reagent (Invitrogen, USA), and then reversely transcribed to complementary DNA via the PrimeScript kit (TaKaRa, China) according to the manufacturer's protocol^42^. The SYBR® Green kit (Bio-Rad, USA) was employed to detect gene expression using the Step-One Plus System. The gene primer sequences were listed in detail in Table 2. The 2^−ΔΔCT^ method was used to determine the relative expression level of mRNA with GAPDH as an internal control.

**Table 2:** The sequences of primers used for qRT-PCR analysis in our study.

Target gene	Sequences of the primers
TRIM31	Sense: 5ʹ-AACCTGTCACCATCGACTGTG-3ʹ Antisense: 5ʹ-TGATTGCGTTCTTCCTTACGG-3ʹ
Axin1	Sense:5ʹ-GAAGACGGCGATCCATCG-3ʹ Antisense: 5ʹ-GGATGCTCTCAGGGTTCT-3ʹ
cyclinD1	Sense: 5ʹ-CCCTCGGTGTCCTACTTCA-3ʹ Antisense: 5ʹ-CTCCTCGCACTTCTGTTCCT-3ʹ
c-Myc	Sense: 5ʹ-CGTAGTTGTGCTGATGTGTGG-3ʹ Antisense: 5ʹ-CTCGGATTCTCTGCTCTCCTC-3ʹ
MMP9	Sense: 5ʹ-GAACCAATCTCACCGACAGG-3ʹ Antisense: 5ʹ-CCACAACTCGTCATCGTCG-3ʹ
GAPDH	Sense: 5ʹ-GGCTGCTTTTAACTCTGGTA-3ʹ Antisense: 5ʹ-ACTTGATTTTGGAGGGATCT-3ʹ

### Colony formation assays

Approximately 500 indicated cells were placed in six-well plates with the complete medium and cultured until colonies were visible, as previously described^22^. The colonies were fixed with 4% formaldehyde for approximately 30 min and stained with Giemsa for 20 min. ImageJ was used to count the number of colonies. These assays were conducted at least thrice.

### Wound healing assays

Approximately 4.0 × 10^5^ cells per well were implanted into a six-well plate until fully confluent, as described previously^30^. The cell layers were scratched using 20 µL sterile pipette tips. The plates were then washed with PBS and cultured in a medium with 5% FBS. Typical images were captured at 0 and 48 h to measure the wound distance after scratching to evaluate the wound closure percentage. Closure rate (100%) = [wound distance (0 h) − wound distance (48 h)]/wound distance (0 h) × 100%. The experiments were performed at least twice.

### Transwell assays

First, the transfected cells were trypsinized, and 3 × 10^3^ cells were suspended in a serum-free medium. Matrigel gel BD (Biosciences) was used in the chambers (8 μm; Corning, Inc.) GC cells were seeded into the upper chambers, and the lower chambers contained 500 μL of medium with 15% FBS as a chemical attractant. After incubation for indicated time points, the migrated and invasive cells were fixed and stained according to a previously published method^28^. Invasive cells were counted and photographed in at least five randomly-selected microscopic fields. The assays were performed in triplicate.

### Vectors and shRNAs transfection

SiRNA targeting Axin1 and shRNAs targeting TRIM31, along with the corresponding control siRNA and scramble shRNA, were both bought from GenePharma (Suzhou, China). The siRNA and shRNA sequences were as follows: Axin1 siRNA (5′-GGACAUGGAUGAGGACGAUTT-3′), TRIM31 shRNA-1 (5′-TATGATGGACTCATGCCTTGC-3′), and TRIM31 shRNA-2 (5′- TTCCCGTCAAAGGAAGTTTGG-3′). The Myc-TRIM31 plasmid was constructed by subcloning the PCR-amplified human TRIM31 coding sequence into the 2 × pcDNA3.1 vector, while Axin1 plasmids were subcloned into the pcDNA3.1 vector with an HA tag. The detailed gene primer sequences were listed in Supplementary Table S1. The GC cells were transfected with the indicated plasmids using TurboFect transfection reagent according to the manufacturer’s protocol (R0532; Thermo Scientific, USA), as previously described^25^.

### Cycloheximide (CHX) chase assay

CHX assays were performed to evaluate the protein stability of Axin1 as previously described^43^. Designated cells were harvested after transfection for 36 h, and treated with the CHX reagent (100 μg/ml) at the indicated time points. Lysates were assessed using western blotting. Half-time decay curves were drawn using the GraphPad Prism software.

### In vivo ubiquitination assay

HGC-27 cells were seeded into 10 cm plate and transfected with scramble shRNA or TRIM31 shRNAs. Upon 48 h, the cells were treated with MG132 for 6 h before harvested. Then indicated cell were lysed by RIPA buffer, and immunoprecipitated with Ni–NTA beads. The ubiquitination levels of Axin1 protein were detected using western blotting assays.

### Co-immunoprecipitation (Co-IP) assays

To investigate the exogenous interaction of Myc-TRIM31 and HA-Axin1, 800–1000 μg of total proteins were mixed with anti-HA beads (CLH104AP; Cedarlane) at 4 °C for 2 h. The beads were washed with IP lysis buffer for more than 3 times and denatured with SDS-PAGE protein loading buffer. Bound proteins were detected by western blotting analysis with anti-Myc (sc-40, Santa Cruz) or anti-HA (sc-7392, Santa Cruz) antibodies described in the figure legends.

### Statistical analysis

Biological assays were independently conducted at least twice. All data were displayed as the mean ± standard deviation. The Kaplan–Meier plotter was used to plot and calculate the survival rate via the log-rank test. The analyses of ROC and time-dependent ROC were made using the R software (version 4.2.1) package pROC (version 1.17.0.1). T-test or one-way analysis of variance (ANOVA) was used for statistical analyses of two or more groups. Chi-square tests were utilized to analyze the correlation between TRIM31 expression and clinicopathological parameters of GC patients. The Pearson correlation test was used for examining correlation analysis of TRIM31 and Axin1 expression in GC tissues. Statistical analysis was performed using R or SPSS software (version 22.0; IBM Corp., Armonk, USA) with *P < 0.05 and **P < 0.01 as statistically significant.

### Ethics approval and consent to participate

The informed consents were obtained from all participants and the acquisition of human tissues used in our study was approved by the Ethics Committee of the Third Affiliated Hospital of Nanchang University (No: KY2022021). This study conforms to the provisions of the Declaration of Helsinki.

### Supplementary Information

Supplementary Information 1.

Supplementary Information 2.

## Data Availability

All data used in this work are included in the article.
