# Supplementary material for: Tripartite motif 31 drives gastric cancer cell proliferation and invasion through activating the Wnt/β-catenin pathway by regulating Axin1 protein stability
Source: Sci Rep. 2023 Nov 16;13:20099. doi: 10.1038/s41598-023-47139-z (PMC10654727; doi:10.1038/s41598-023-47139-z)
Supplement: Supplementary file 1 — Supplementary Information 1. [file 41598_2023_47139_MOESM1_ESM.pdf]

Tripartite motif 31 drives gastric cancer cell proliferation and invasion through activating the Wnt/β-catenin pathway by regulating Axin1 protein stability

Qi Feng[1#], Fengting Nie[2#], Lihong Gan[1#], Xianpin Wei[2#], Peng Liu[1], Hui Liu[1], Kaige Zhang[1], Ziling Fang[2*], Heng Wang[3*] and Nian Fang[1*]

[1]Department of Gastroenterology, the Third Affiliated Hospital of Nanchang University or Nanchang First Hospital, 128 Xiangshan North Road, Nanchang, Jiangxi Province, P.R. China

[2]Department of Oncology, the First Affiliated Hospital of Nanchang University, 1519 Dongyue Avenue, Nanchang, Jiangxi Province, P.R. China

[3]Department of Orthopedics, the First Affiliated Hospital of Nanchang University, 1519 Dongyue Avenue, Nanchang, Jiangxi Province, P.R. China

Supplementary Files

Figures S1, Figure S2, Figure S3, Figure S4 and Figure S5

Table S1

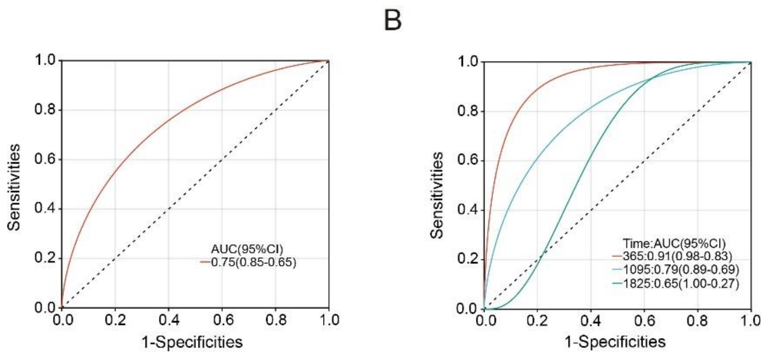

Figure Supplementary 1. The analysis for ROC and time-dependent ROC of TRIM31 overexpression. (A) The ROC analysis of TRIM31 overexpression in GC patients. (B) The time-dependent ROC analysis (at 1, 3, 5 years) of TRIM31 expression.

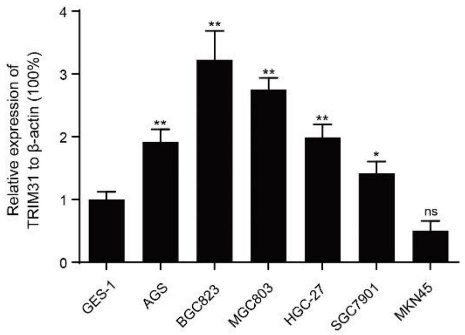

Figure Supplementary 2. Bar graph of quantified relative TRIM31 expression normalized to β-actin expression in GES-1 and six GC cell lines by western blotting (*P < 0.05 and **P < 0.01).

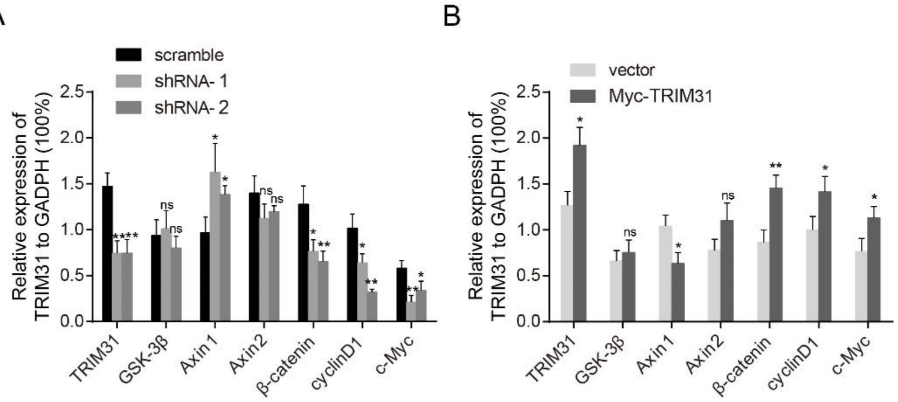

Figure Supplementary 3. Relative protein expression of core Wnt/β-catenin pathway components and TRIM31 after indicated transfection by western blotting. (**A**) Bar graph of quantified relative gene expression upon TRIM31 knockdown. (**B**) Bar graph

of quantified relative gene expression upon TRIM31 overexpression (*P < 0.05, **P < 0.01, and ns means no significance).

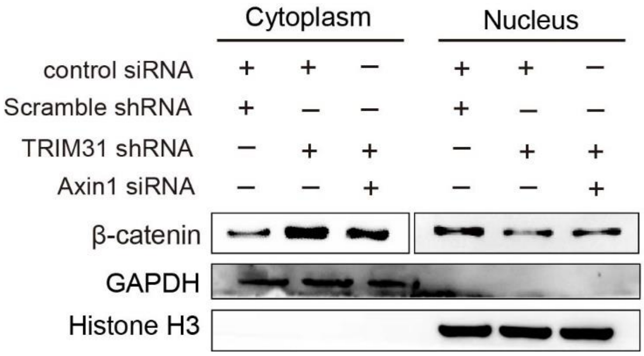

Figure Supplementary 4. The cytoplasmic/nuclear fractionation of β-catenin protein was analyzed through western blotting assays upon transfection

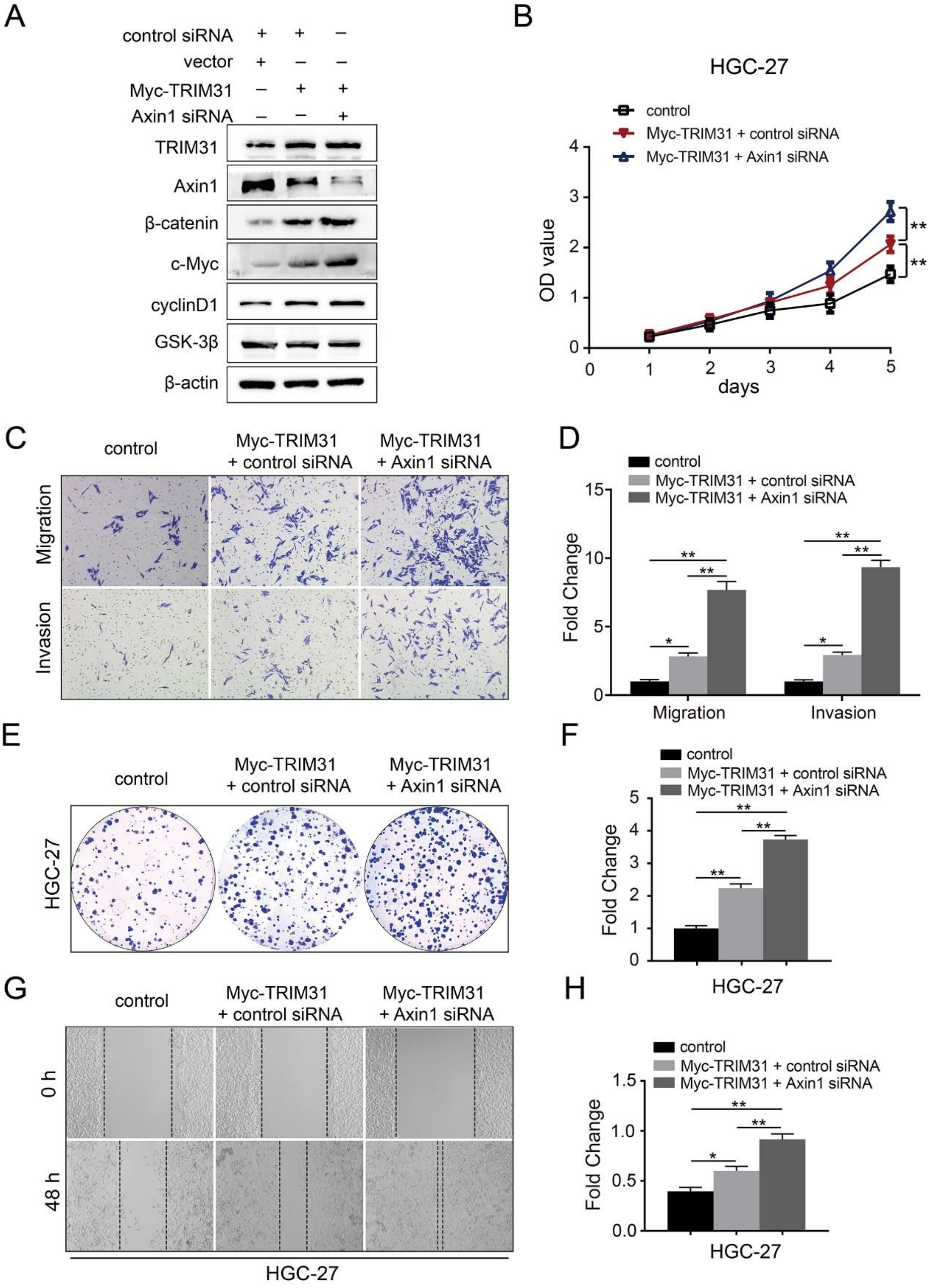

Figure Supplementary 5. Axin1 silencing promoted the proliferation and invasion of TRIM31 overexpression in GC cells. (**A**) Relative protein expression levels of TRIM31, Axin1, and main Wnt pathway components were analyzed through western blotting assays upon transfection. (**B**) OD values were detected by CCK-8 assays in indicated HGC-27 cells. (**C, D**) Representative images of modified HGC-27 from transwell

migration and invasion analysis (magnification, ×200). Bar graphs of the migration and invasion data. (**E, F**) Representative images and relative quantification of HGC-27 cells from colony-formation experiments. (**G, H**) Wound healing assays showed the migratory capabilities of modified GC cells. The relative quantification was shown in the bar graph (One-way ANOVA: *$P < 0.05$ and **$P < 0.01$).

Target Gene	Sequences of the Primers
pcDNA3.1-Myc-TRIM31	Sense: 5'-CCCAAGCTTGCCGCCACCATGGAGCAGAAGCTGATCTCAGAGGAGGACCTGATGGCCAGTGGGCAG-3'<br>Antisense: 5'-CCGGAATTCTTAGCTTGAAGGAACCTC-3'
pcDNA3.1-HA-Axin1	Sense: 5'-GCTTGGTACCGAGCTCGGATCCGCCACCATGTACCCATACGATGTTCCAGATTACGCTAATATCCAAGAGCAGGGTTTCCCCTTGGAC-3'<br>Antisense: 5'-TGCTGGATATCTGCAGAATTCTCAGTCCACCTTCTCCACTTTGCCGATGATCTTCTCCTCAAAGACG-3'

Supplementary Table S1. The oligomer sequences are listed in our study.
