# Supplementary material for: Tripartite motif 31 drives gastric cancer cell proliferation and invasion through activating the Wnt/β-catenin pathway by regulating Axin1 protein stability
Source: Sci Rep. 2023 Nov 16;13:20099. doi: 10.1038/s41598-023-47139-z (PMC10654727; doi:10.1038/s41598-023-47139-z)

Tripartite motif 31 drives gastric cancer cell proliferation and invasion through activating the Wnt/β-catenin pathway by regulating Axin1 protein stability

Qi Feng[1#], Fengting Nie[2#], Lihong Gan[1#], Xianpin Wei[2], Peng Liu[1], Hui Liu[1], Kaige Zhang[1], Ziling Fang[2*], Heng Wang[3*] and Nian Fang[1*]

[1]Department of Gastroenterology, the Third Affiliated Hospital of Nanchang University or Nanchang First Hospital, 128 Xiangshan North Road, Nanchang, Jiangxi Province, P.R. China

[2]Department of Oncology, the First Affiliated Hospital of Nanchang University, 1519 Dongyue Avenue, Nanchang, Jiangxi Province, P.R. China

[3]Department of Orthopedics, the First Affiliated Hospital of Nanchang University, 1519 Dongyue Avenue, Nanchang, Jiangxi Province, P.R. China

Original images of the blots

Figure 2D

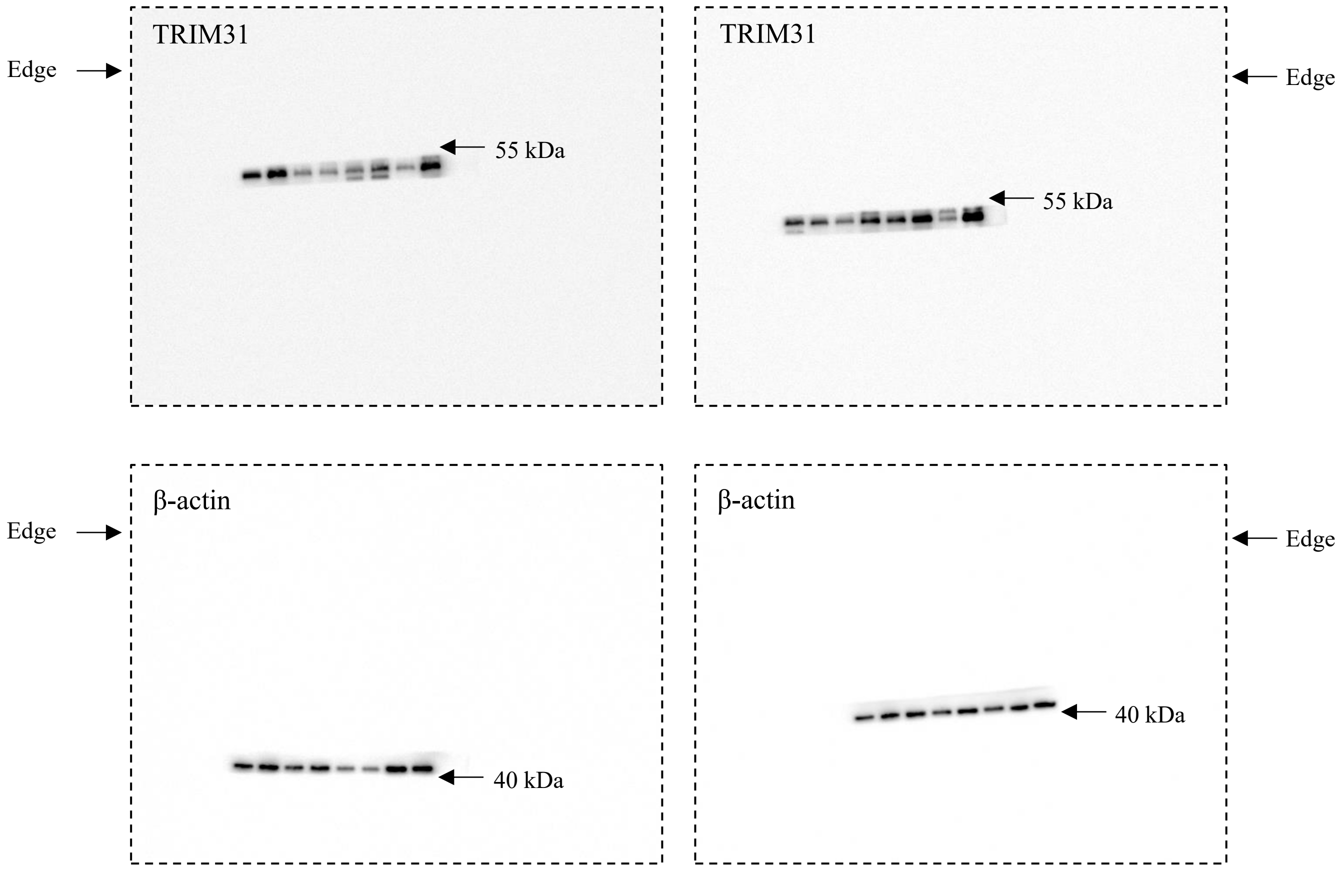

Figure 2F

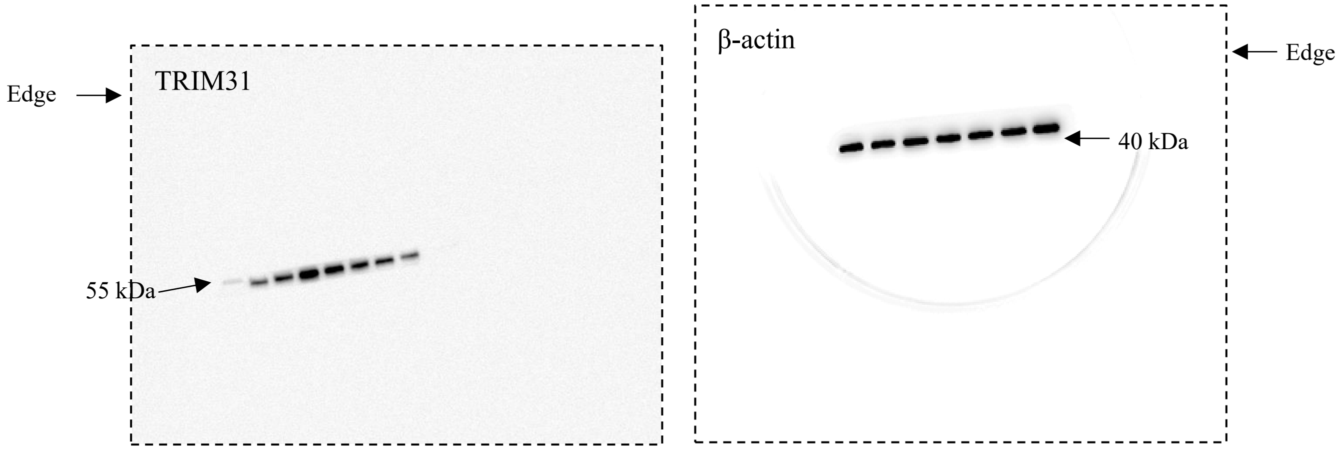

Figure 4A

Edge
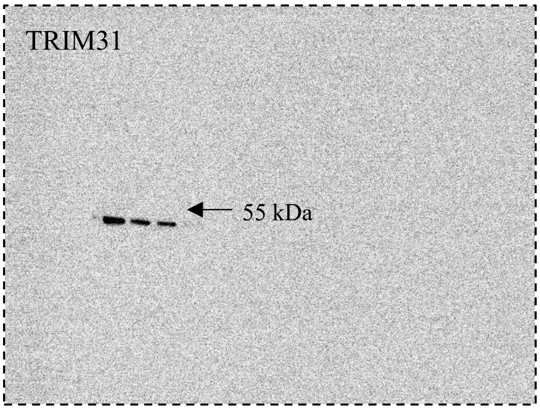

The left picture was replaced duo to overexposure. The right one was corresponding internal control.

Edge
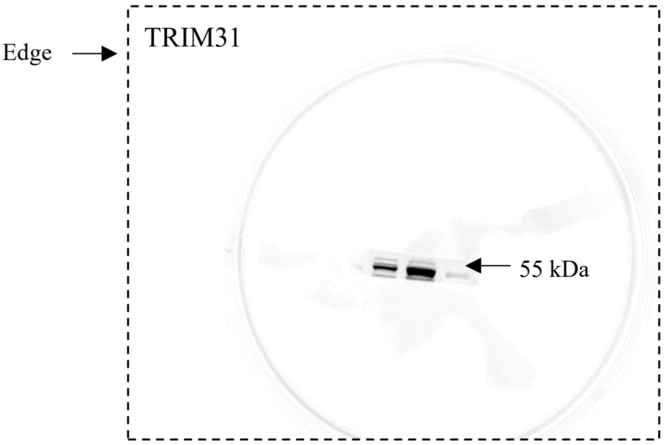

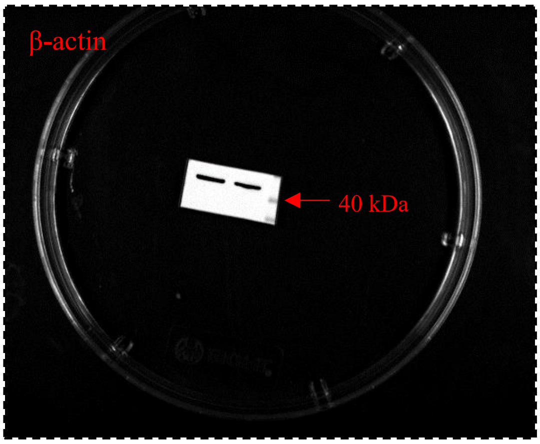

Edge

Edge
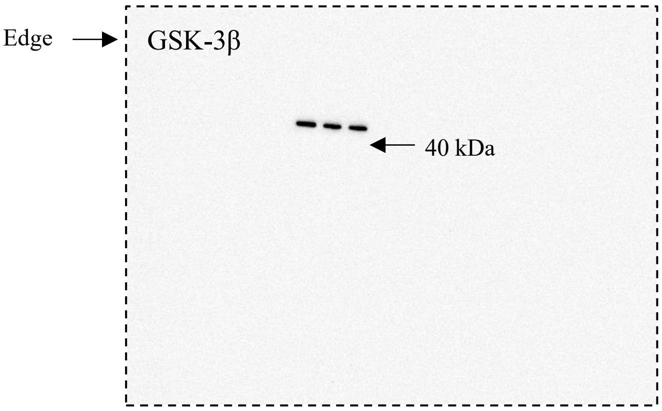

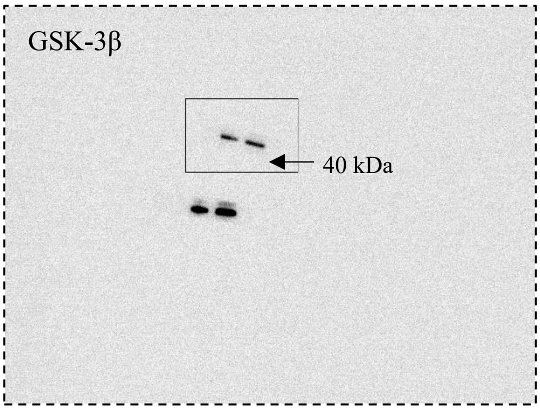

Edge

Figure 4A

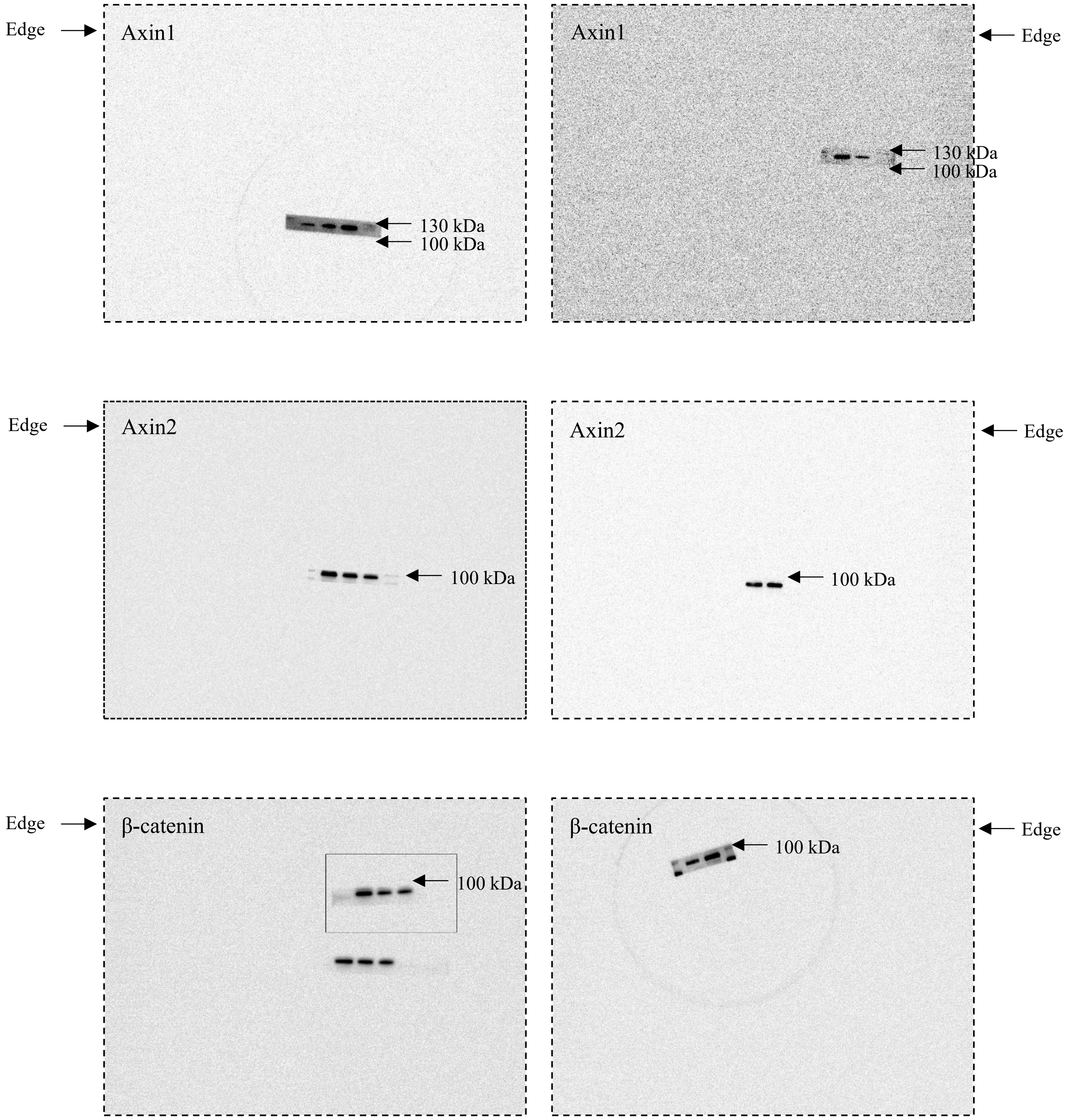

Edge
Axin1
130 kDa
100 kDa
Axin1
Edge
130 kDa
100 kDa
Edge
Axin2
100 kDa
Axin2
Edge
100 kDa
Edge
β-catenin
100 kDa
β-catenin
Edge
100 kDa

Figure 4A

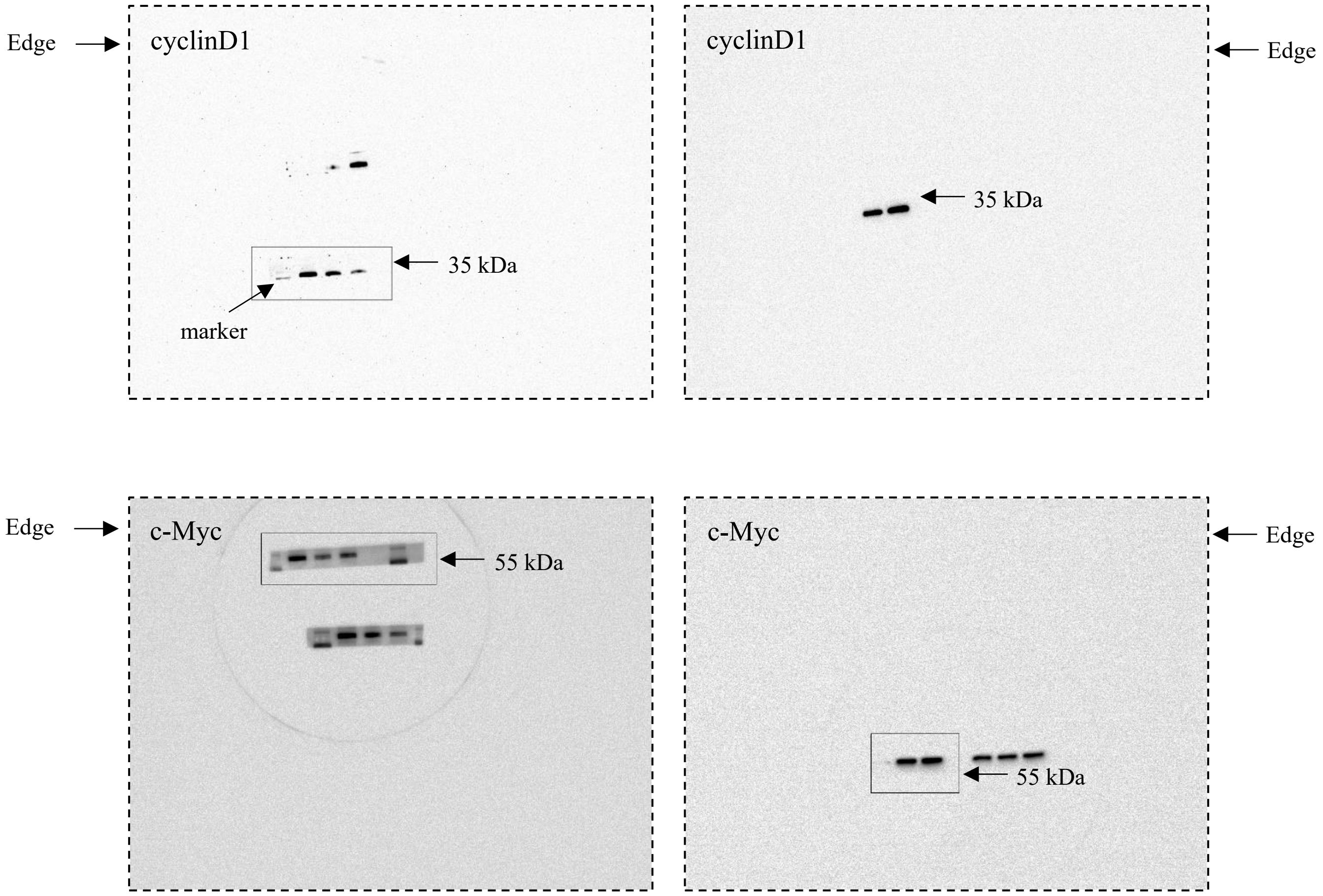

The left picture was replaced as follows duo to overexposure.

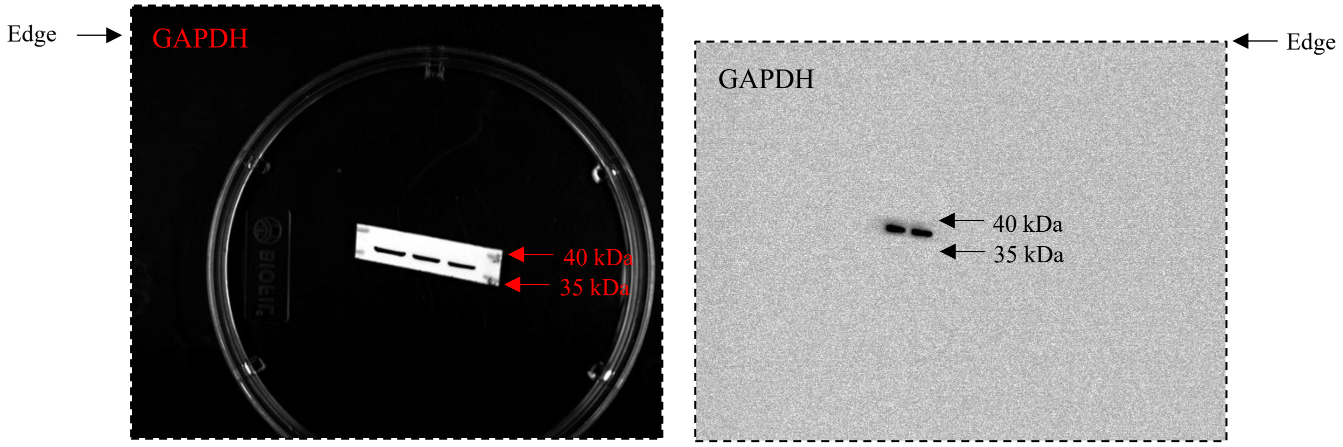

Figure 4B

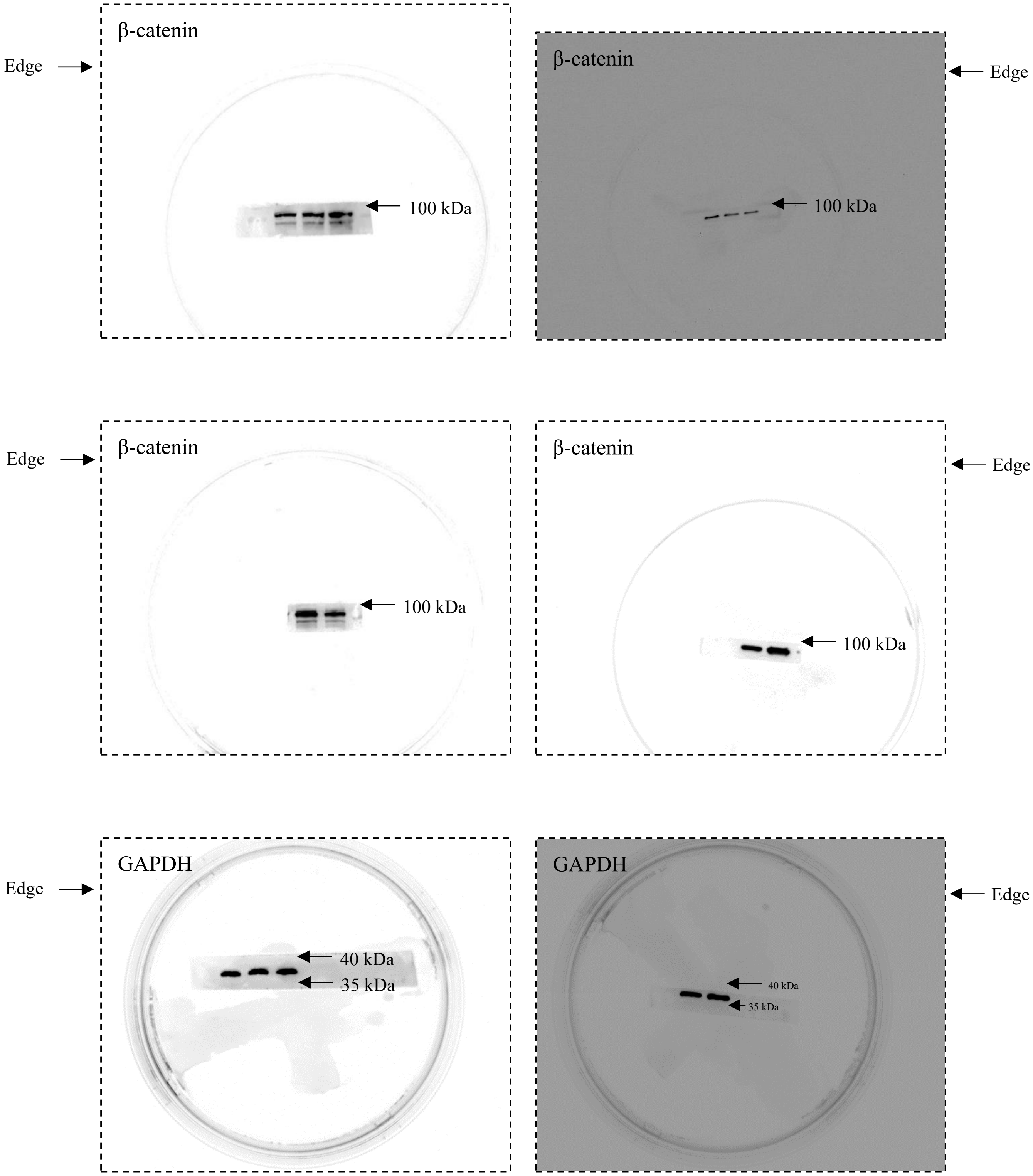

β-catenin
Edge
100 kDa
β-catenin
Edge
100 kDa
β-catenin
Edge
100 kDa
β-catenin
Edge
100 kDa
GAPDH
Edge
40 kDa
35 kDa
GAPDH
Edge
40 kDa
35 kDa

Figure 4B

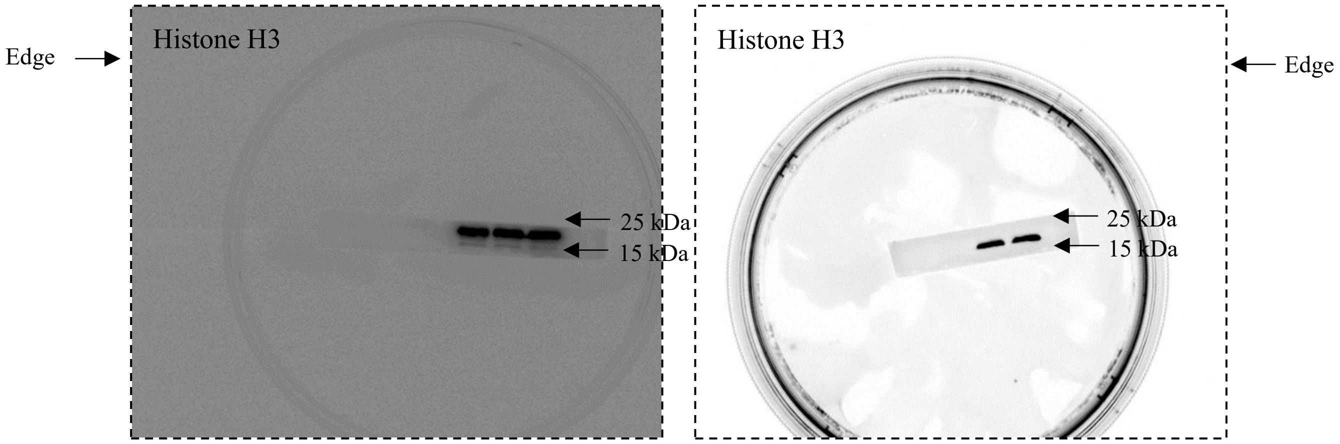

Figure 4E

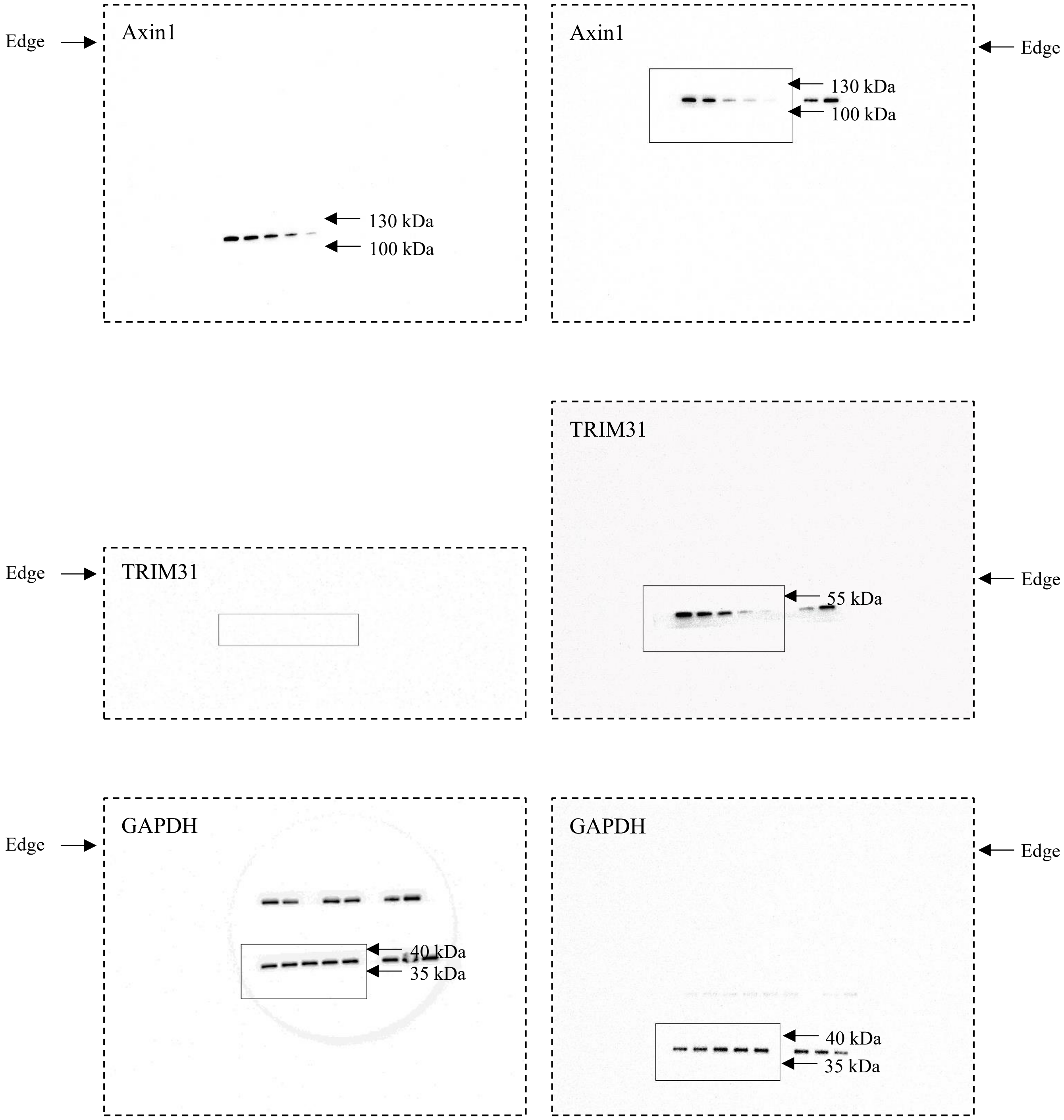

Edge
Axin1
130 kDa
100 kDa
Axin1
130 kDa
100 kDa
Edge
TRIM31
Edge
TRIM31
55 kDa
Edge
GAPDH
Edge
40 kDa
35 kDa
GAPDH
40 kDa
35 kDa
Edge

Figure 4G

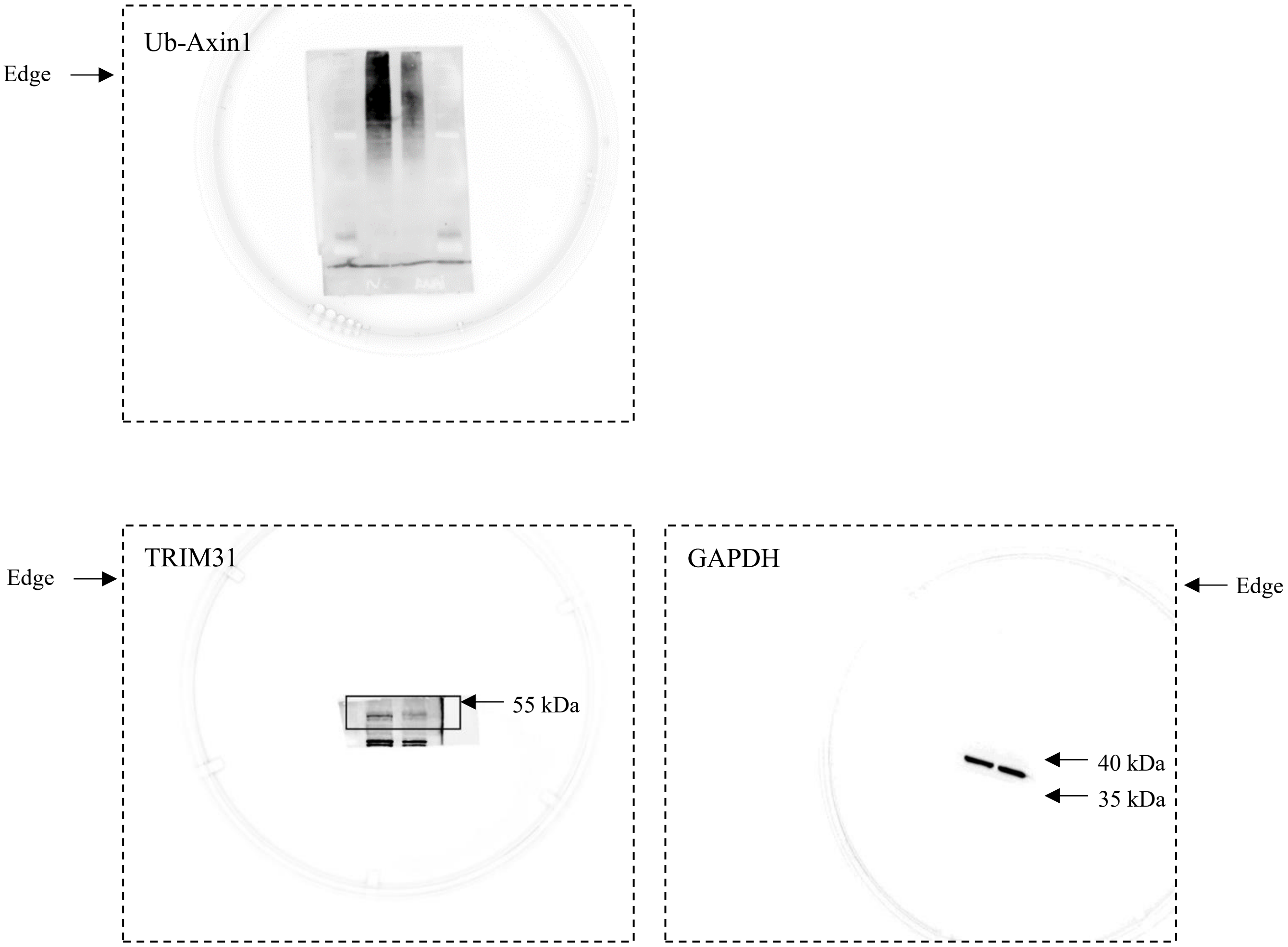

Figure 4H

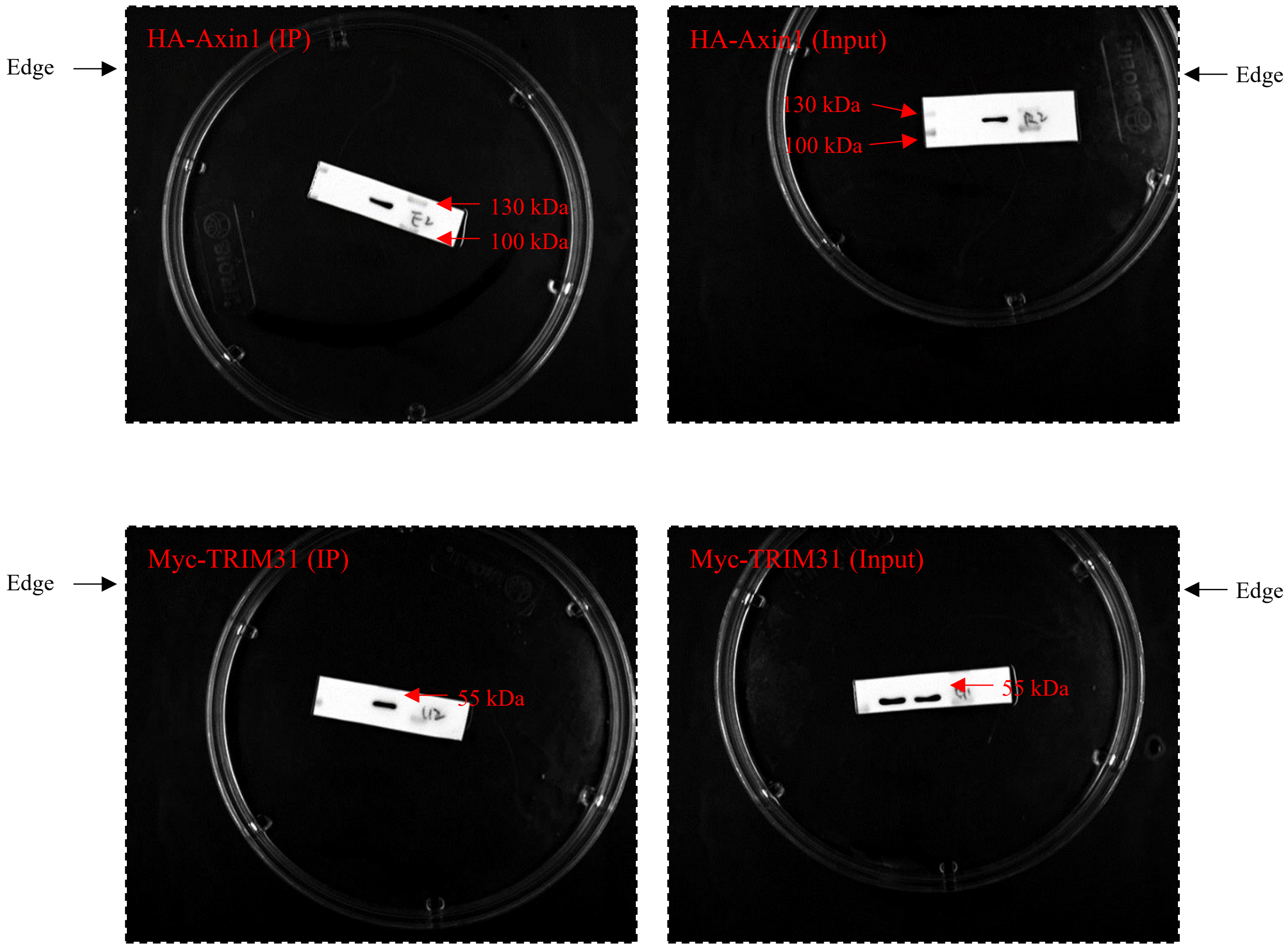
HA-Axin1 (IP)
130 kDa
100 kDa
HA-Axin1 (Input)
130 kDa
100 kDa
Myc-TRIM31 (IP)
55 kDa
Myc-TRIM31 (Input)
55 kDa
Edge
Edge
Edge
Edge

Figure 5A

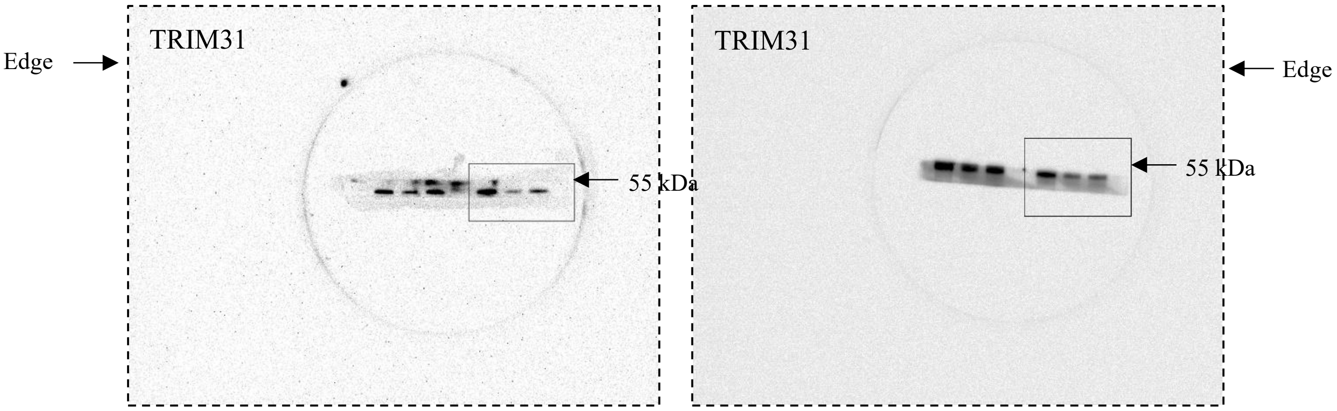

The left picture was replaced duo to overexposure. The right one was corresponding internal control.

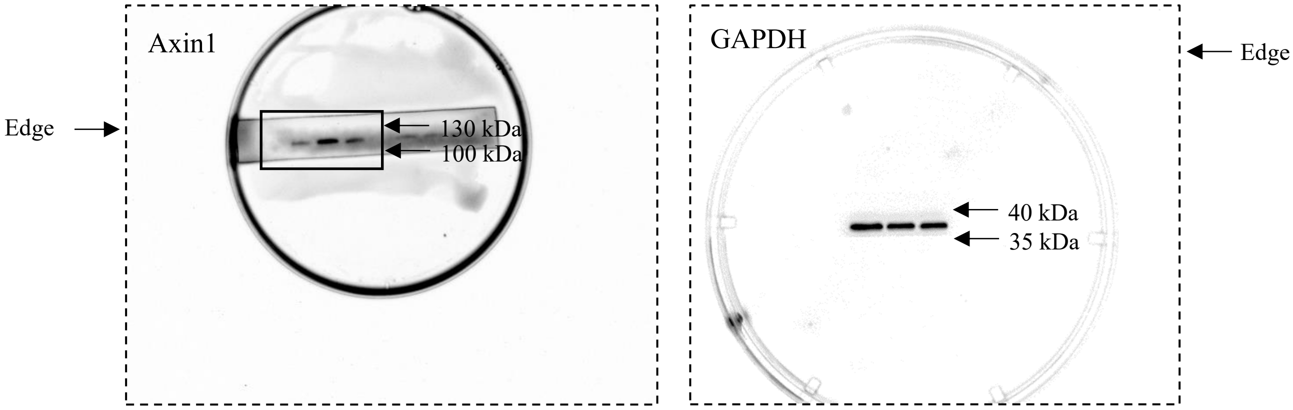

The left picture was replaced duo to overexposure. The right one was corresponding internal control.

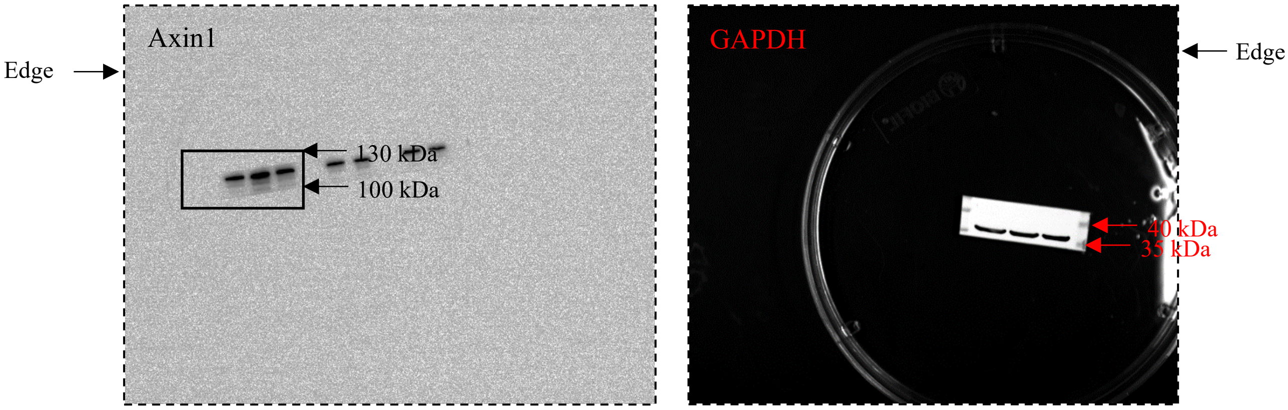

Figure 5A

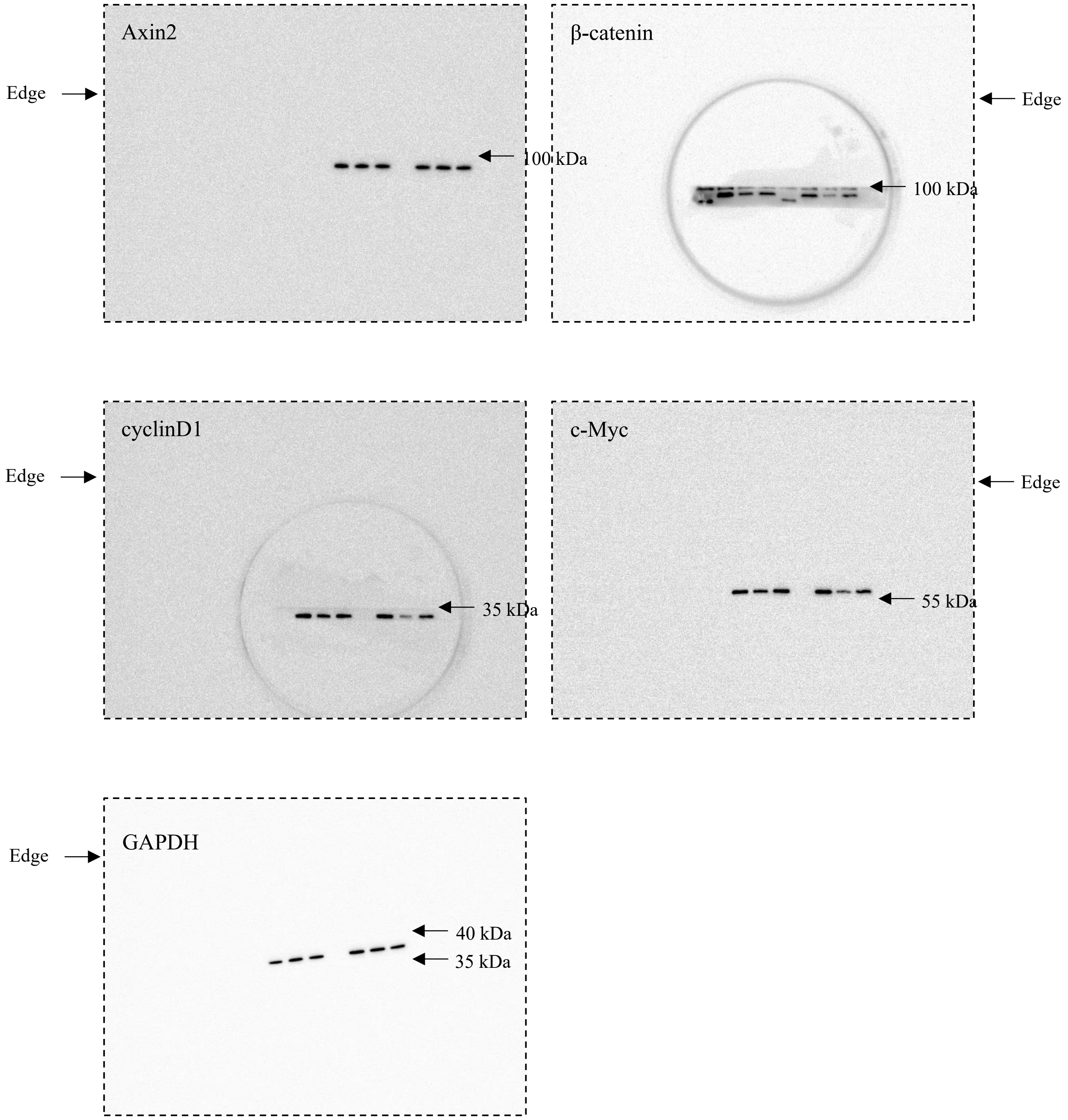

Axin2
Edge
100 kDa
β-catenin
Edge
100 kDa
cyclinD1
Edge
35 kDa
c-Myc
Edge
55 kDa
GAPDH
Edge
40 kDa
35 kDa

Figure 6

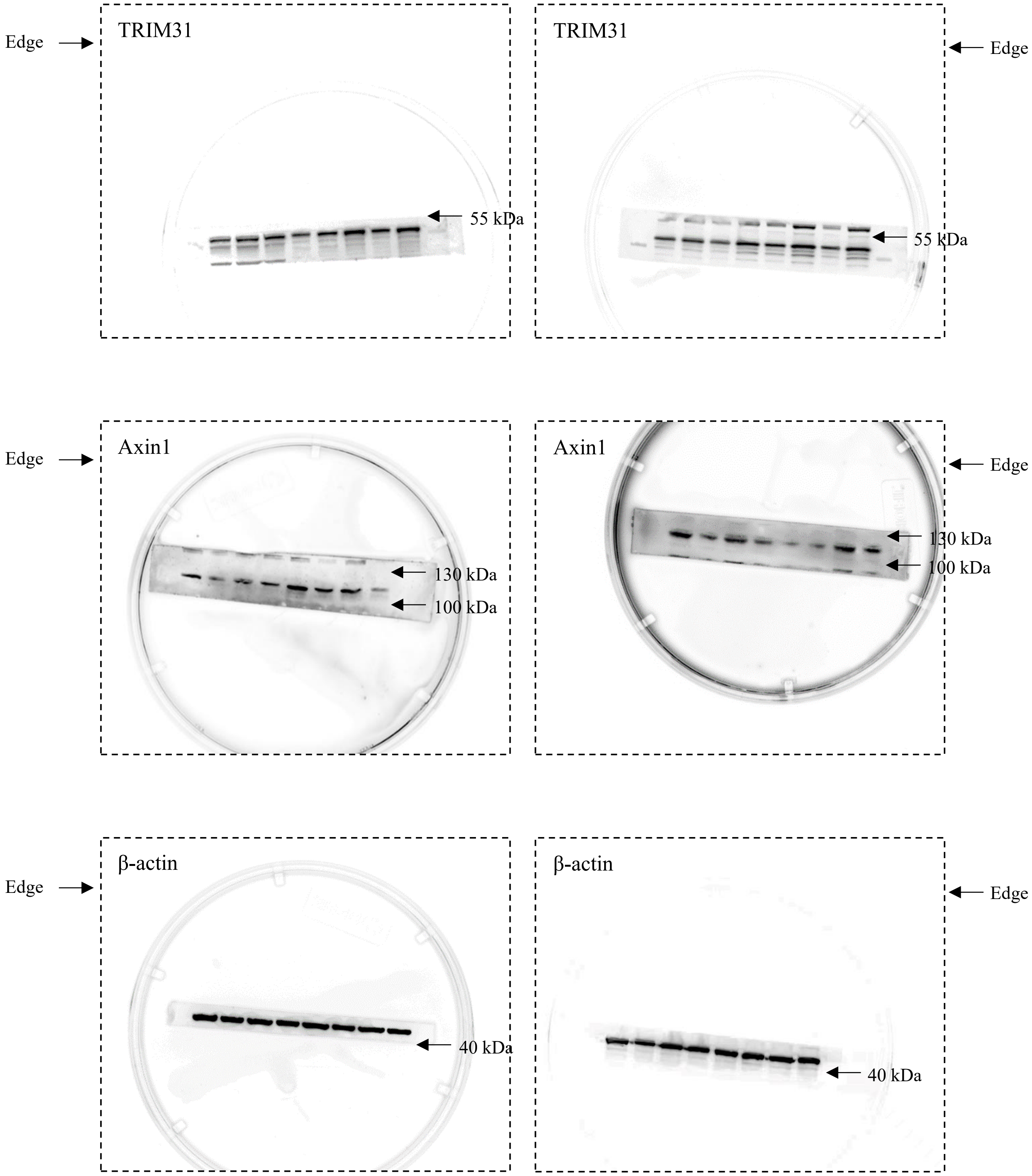
Edge
TRIM31
55 kDa
TRIM31
Edge
55 kDa
Edge
Axin1
130 kDa
100 kDa
Axin1
Edge
130 kDa
100 kDa
Edge
β-actin
40 kDa
β-actin
Edge
40 kDa

Supplementary Figure 3

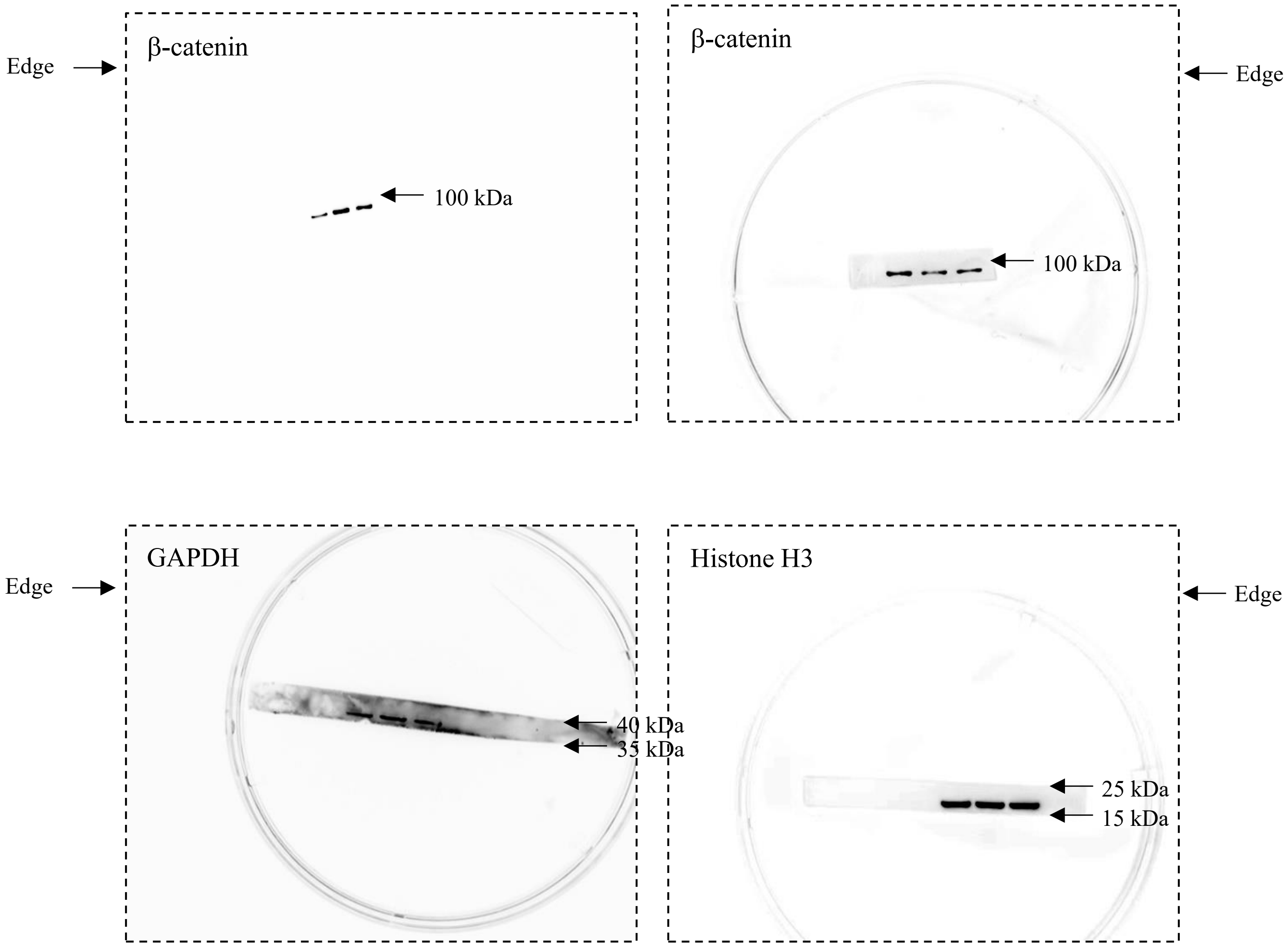

Supplementary Figure 4A

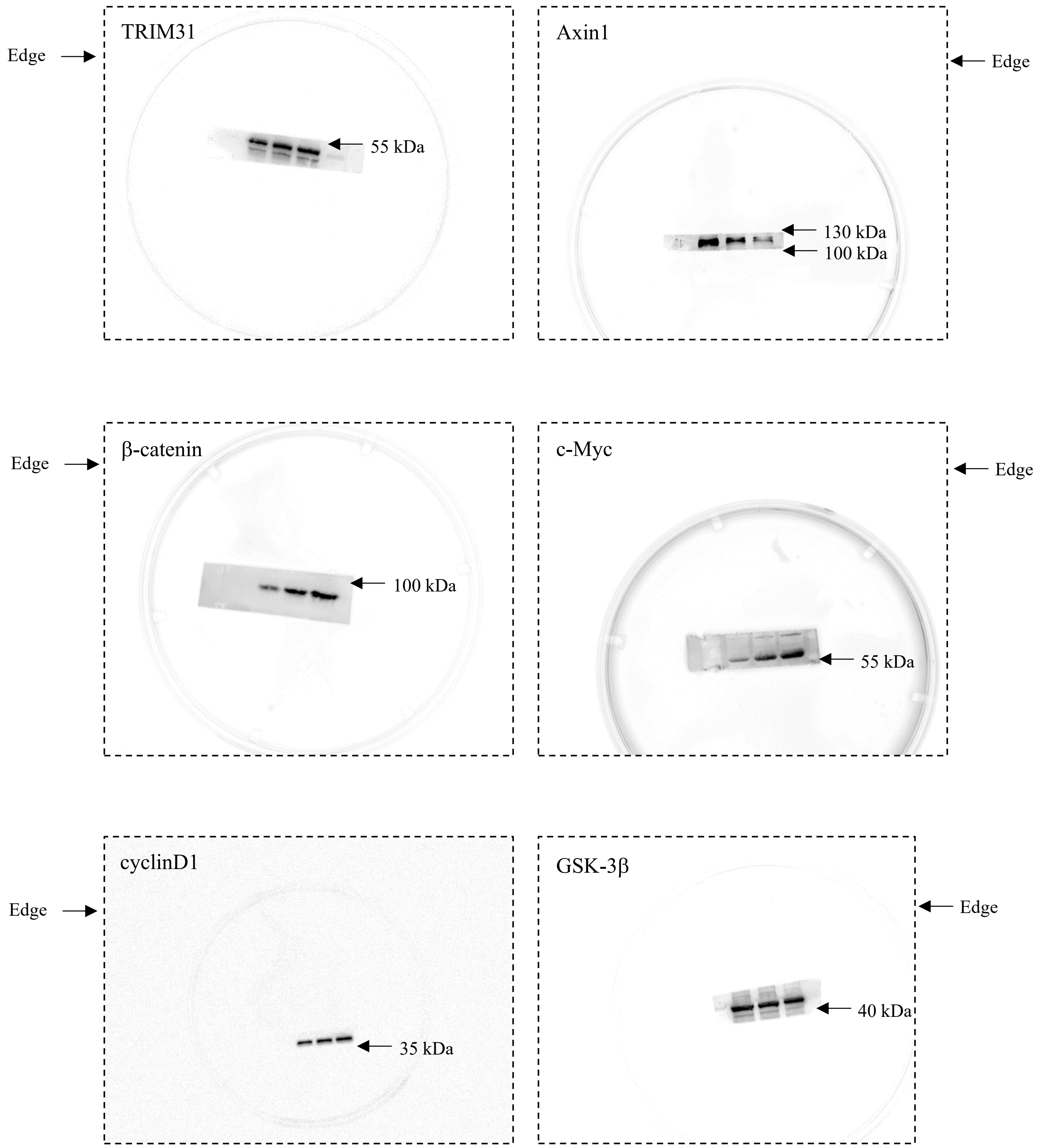

Supplementary Figure 4A

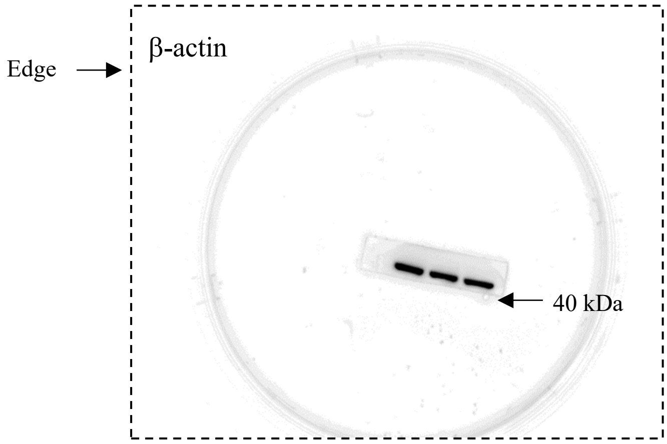

Supplement: Supplementary file 2 — Supplementary Information 2. [file 41598_2023_47139_MOESM2_ESM.pdf]
